# Bioprinting of gelatin-based materials for orthopedic application

**DOI:** 10.3389/fbioe.2024.1357460

**Published:** 2024-03-13

**Authors:** Yusuf Olatunji Waidi, Ishaq Kariim, Sudipto Datta

**Affiliations:** ^1^ Indian Institute of Science (IISc), Bangalore, India; ^2^ Nelson Mandela African Institution of Science and Technology, Arusha, Tanzania; ^3^ Chemical Engineering Department, Federal University of Technology, Minna, Nigeria

**Keywords:** bioprinting, gelatin, bioinks, scaffolds, bone, tissue engineering

## Abstract

Bio-printed hydrogels have evolved as one of the best regenerative medicine and tissue engineering platforms due to their outstanding cell-friendly microenvironment. A correct hydrogel ink formulation is critical for creating desired scaffolds that have better fidelity after printing. Gelatin and its derivatives have sparked intense interest in various biomedical sectors because of their biocompatibility, biodegradability, ease of functionalization, and rapid gelling tendency. As a result, this report emphasizes the relevance of gelatin-based hydrogel in fabricating bio-printed scaffolds for orthopedic applications. Starting with what hydrogels and bio-printing are all about. We further summarized the different gelatin-based bio-printing techniques explored for orthopedic applications, including a few recent studies. We also discussed the suitability of gelatin as a biopolymer for both 3D and 4D printing materials. As extrusion is one of the most widely used techniques for bio-printing gelatin-based, we summarize the rheological features of gelatin-based bio-ink. Lastly, we also elaborate on the recent bio-printed gelatin-based studies for orthopedics applications, the potential clinical translation issues, and research possibilities.

## Graphical Abstract

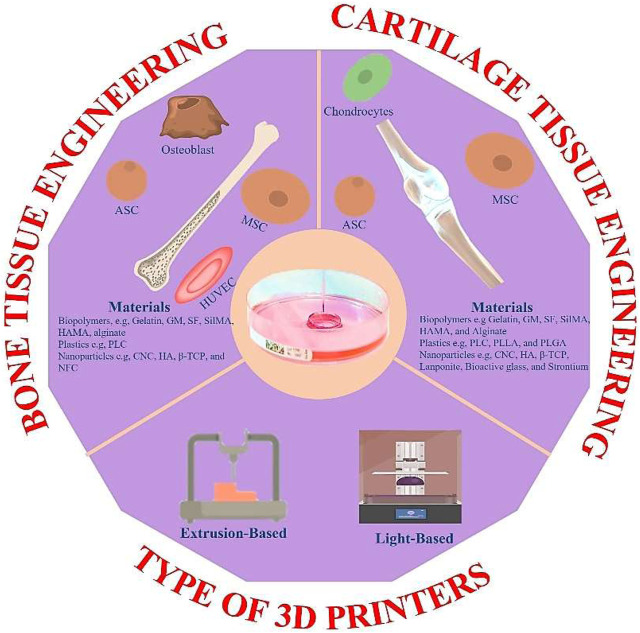

## 1 Introduction

Restoring a significant large bone or cartilage defect caused by illnesses, accidents, or trauma is one of the most challenging tasks in orthopedic therapeutic settings (Agarwal et al., 2021; Hu et al., 2024; Huang et al., 2024; Martins et al., 2024). While the regeneration of orthopedic tissue is a gradual process, it is also a complicated one that necessitates a wide range of abilities and knowledge. In the past, despite numerous attempts to resolve these difficulties. However, the present success is very little. The existing techniques have several disadvantages, including sluggish bio-integration and a significant risk of infection (Moriarty et al., 2019; Hou et al., 2024; Wu et al., 2024). Providing a close and comparable 3D milieu to natural tissue is critical to repairing faulty bone and cartilage, along with significant cell attachment, proliferation, differentiation, and the required bioactive component (Sood et al., 2021). Bioprinting has lately evolved as the best method for producing patient-specific cell-laden scaffolds for tissue regeneration, such as bone and cartilage. Bio-printing poses a significant edge to traditional fabricating approaches regarding scaffold geometry control, scaffold dimension specificity to properly match the intended place, 3D scaffold microstructures environment, and encapsulated cells’ good spatiotemporal dispersion (Sun et al., 2020; Cheng et al., 2021).

The classifications of 3D printing techniques in tissue engineering applications include 1) cell seeding on an already printed porous volume for cell adherence and proliferation to achieve an engineered tissue and 2) mixing cells with a bio-friendly ink before 3D printing to achieve better interactions and control of cells with their microenvironment. However, different strategies have distinct benefits and drawbacks. Among the bioprinting methods, the extrusion-based process of bioprinting stands out due to its cost-effectiveness, simplicity, and scalability, and thus, it is the most often employed (Cheng Y. et al., 2019; Chen et al., 2023). Appropriate rheological qualities, such as shear thinning behavior and thixotropy properties, are critical with this approach because they govern the extruding manner via a nozzle under pressure and the ability to preserve the intended form post-deposition (Li et al., 2019). As a result, it is critical to engineer a functional hydrogel ink with desired rheological and mechanical characteristics (Gopinathan and Noh, 2018; Gao et al., 2019). Furthermore, the bio-inks must be biocompatible, biodegradable, and tissue-specific without adversely affecting the encapsulated cells and adjacent tissues.

Hydrogels are a class of 3D network polymers with enormous capacity to expand and retain a high-water quantity. Recently, hydrogels have emerged as the most widely used material to make 3D-printed constructs for various tissue engineering applications. Painfully, hydrogels’ poor degradation rate and weak mechanical characteristics restrict their use as bone tissue engineering (BTE) biopolymers. Gelatin is an intriguing biomaterial for hydrogel formulation among natural polymers because of its gelation capabilities (Cheng et al., 2018; Choi et al., 2018; Yan et al., 2018; Nabavinia et al., 2019). As a low-cost polymer, gelatin offers good biomaterial features such as biodegradability, biocompatibility, cell adherence, and proliferation. In addition, gelatin is generally produced by mild hydrolysis of collagen (Saravanan et al., 2017; Hasan et al., 2018; Kim et al., 2018). It has also been used with other biomaterials to increase cell adhesion (Piao and Chen, 2017). Gelatin possesses exceptional biological properties due to the RGD (Arg-Gly-Asp) sequence, making it a superior choice for BTE to other biopolymers. Furthermore, it stimulates osteoclasts, contributing to enhanced osteogenesis (Kruppke et al., 2020; Wu et al., 2020). Despite its numerous biomaterial features, gelatin’s mechanical qualities and rapid breakdown restrict its application as a hard tissue engineering material (Hasan et al., 2018; Nabavinia et al., 2019). Adding reinforcing components to form a composite material is a valuable method for improving the characteristics of materials. Several researchers have recently published various research articles on bio-nanocomposites for orthopedics applications. These bio-nanocomposite bone replacements usually include granules, powders, porous or solid constructs, and bioactive metal prosthesis coatings (Yekta et al., 2018; Shuai et al., 2018; Shuai et al., 2019a; Shuai et al., 2019b; Kordjamshidi et al., 2019; Saber-Samandari et al., 2019). Various reinforcing materials have been explored in the past, which includes hydroxyapatite (HAp) (Nabavinia et al., 2019), graphene, and carbon nanotubes (Piao and Chen, 2017; Yan et al., 2018).

**FIGURE 1:**
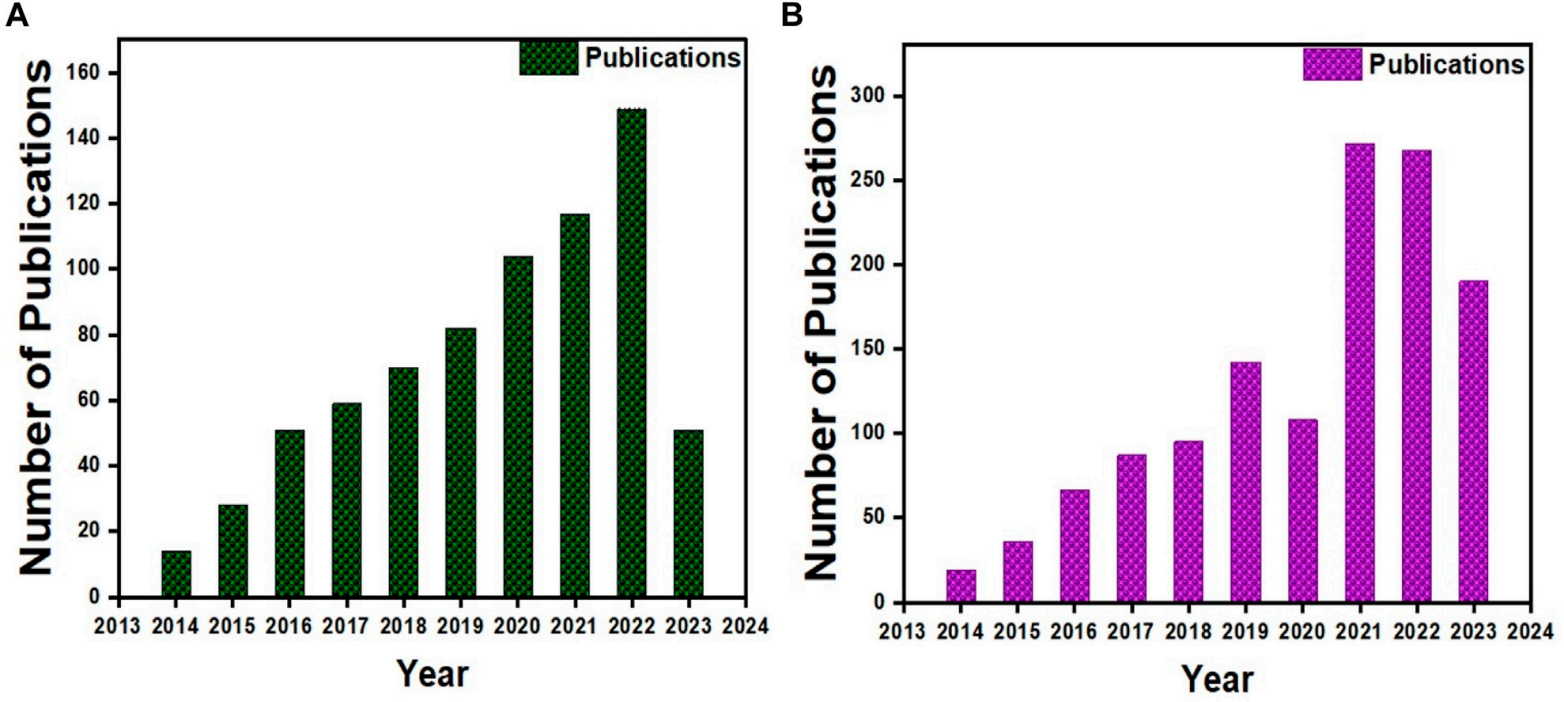
Publication trends for bioprinting **(A)** BTE and **(B)** CTE.

**FIGURE 2:**
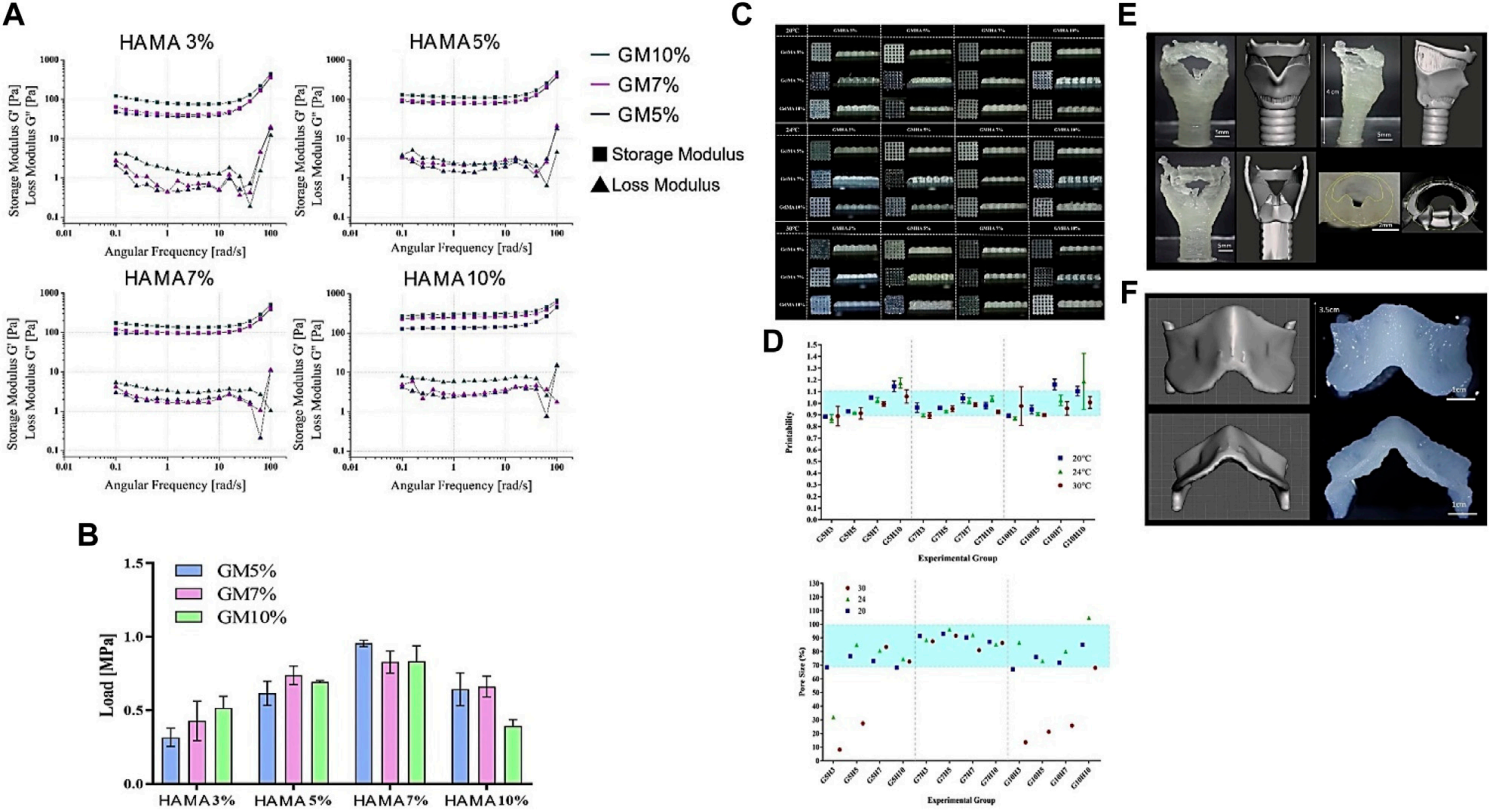
Rheological, Mechanical, and Printability assessment of various bio-ink formulations illustrating **(A)** Viscoelasticity. **(B)** Compressive strength **(C)** GM/HAMA bio-ink 3D printed mesh scaffolds **(D)** Printability measurement using mesh images **(E)** Rabbit larynx CAD model and 3D Printed larynx with G7H5 bio-ink. **(F)** Rabbit thyroid cartilage CAD model and 3D Printed thyroid cartilage with G7H5 bio-ink. Reproduced with permission (Lee et al., 2020).

More than 500 papers have been published for cartilage tissue bioprinting (ref. Scopus search 3D Bio-printing, cartilage) (Figure 2A) and more than 800 for bone (ref. Scopus search 3D Bio-printing, Bone) (Figure 2B). Moreover, half of those publications were reported in the recent 3 years, indicating that it is an active area of research. Gelatin and its derivatives have received a lot of interest among these published studies because of its simplicity of synthesis at a cheap cost, acceptable biocompatibility, transparent structure for cell monitoring, photo-crosslink ability, and customizable physical and chemical characteristics (Xiao et al., 2019). However, few review papers on gelatin-based 3D bioprinting have been published thus far (Wang et al., 2017; Ying et al., 2018). To our knowledge, these review papers only cover a small portion of gelatin derivatives and applications, with no mention of orthopedic uses. Therefore, this review article summarized the most recent advances in bioprinted gelatin-based formulations for orthopedic repair. The interconnectivity summary between the gelatin-based formulations, printability, and biological functions of orthopedic cells. Gelatin-based bio-printing presents status and critical challenges summary to achieve more effective clinical translation. Figure 1 shows the trends for bioprinting of (a) BTE and (b) Cartilage tissue engineering (CTE).

## 2 3D bioprinting methods for gelatin-based inks

Micro molding, the most common method of creating gelatin-based 3D structures, fosters diverse cell activities. However, the bioprinting method has taken over due to the possibility of introducing typical complexity of 3D design, resolution, and spatial control over the fabricating process (Hölzl et al., 2016; Alhaskawi et al., 2024; Bhardwaj et al., 2024). The viscosity of bio-ink is a crucial aspect to consider in bio-printing approaches, particularly extrusion-based techniques (Catros et al., 2011). Pure gelatin bioprinting is difficult due to its intrinsic temperature sensitivity and poor viscosity at ambient temperature or higher. As a result, it could not meet the criterion for various tissues. Because of the various chemical and mechanical characteristics linked with different tissues and to mimic tissue ECM in nature, gelatin-based bio-ink has primarily been used in a modified form. Some of these alterations include concentration optimizations, functionalization with other molecules, and the insertion of second phases into the gelatin matrix. These parameters can also influence bio-ink characteristics, making them suited for specific tissues.

### 2.1 Extrusion based bioprinting

**FIGURE 3:**
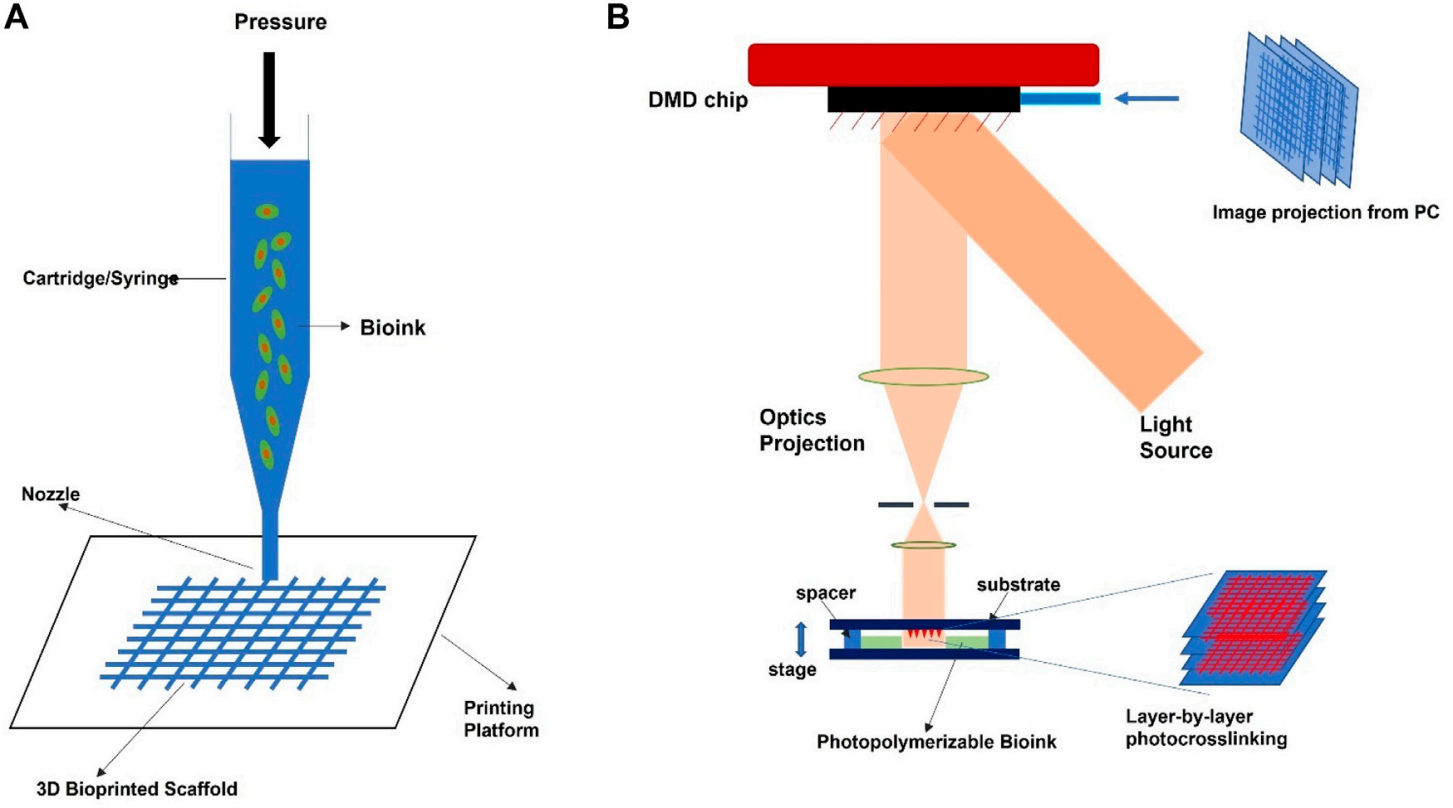
Schematic illustration diagram of common bioprinting technology for orthopedic applications. **(A)** extrusion-based technique, **(B)** light-based technique.

A wide range of 3D bio-printing technologies is widely accessible (Vijayavenkataraman et al., 2018; Vanaei et al., 2021), such as extrusion, inkjet, microfluidic, stereolithography, and laser-based techniques. These techniques enable variability in resolution levels, printing precision, working volumes, acceptable bio-inks, and the capacity to inculcate cells due to their material processing principles. Furthermore, their production speed and total consumption prices differ, resulting in efficiency differences. As a result, the extrusion bioprinting technology appears to be the most promising option for producing therapeutically relevant scaffolds (cm range) (Malda et al., 2013; Herzog et al., 2024; Padhy et al., 2024). Extrusion printing involves layer-by-layer bio-ink deposition on the printing platform through a cartridge nozzle (Figure 3A). However, in the absence of cells, the deposited substance is called biomaterial ink, and with cells, it should be referred to as bio-ink (Groll et al., 2019). Extrusion printing can be either pneumatically or mechanically propelled. In this printing method, various parameters that inform the printing qualities include effective flow rate, printing speed, and rheological qualities of the gel. These bioprinting approaches entail straightforward equipment, simplicity of use, and minimal cost. Another feature that distinguishes this technology is its high manufacturing volume, excellent precision (up to a micrometer level), and tremendous design flexibility (Schwab et al., 2020). Several computer-aided design (CAD) models, which are simple, easy to use, and consist of medical photographs of patients’ specific tissues, are freely or commercially accessible.

Gelatin and its derivatives are often used as hydrogel materials in extrusion bioprinting because they have minimal cell toxicity, strong biocompatibility, high accessibility, excellent rheological characteristics, and easy/flexible handling. However, maintaining good form accuracy and post-printing stability, particularly at physiological temperatures, as well as mechanical qualities that match the intended tissue, can be difficult due to gelatin’s inherent poor mechanical characteristics and temperature reactivity. Solving the latter problem entails altering gelatin with a molecule that imparts chemical or enzymatic crosslinking properties. Such modifications can aid post-printing stability/degradation because pure gelatin only has physical crosslinking depending on temperature. In addition, yield stress is essential in identifying the best biomaterial ink for extrusion printing methods. For example, static yield stress (α_stat_) is necessary for proper gel flow through the nozzle. While the dynamic yield stress (α_dyn_) determines the gel flow regulation. The bio-ink must possess both rigidity to facilitate the continuous filament extrusion through the nozzle in conjunction with minor deformation for shear thinning behavior necessary during extrusion bioprinting (Smith et al., 2018) (Figure 2). Printing gelatin at temperatures much below its melting point is the most popular method due to its gelation and viscous nature at such temperatures, which facilitate the printing handling.

### 2.2 Light based bioprinting

Recently, light-based bioprinting has attracted much interest in the biomedical field, especially tissue engineering, due to its suitability to generate complex tissue architectures. The commonly available light-based bioprinting techniques include mask-based stereolithography (SLA), laser-based SLA, and digital light processing (DLP). DLP stands out among all these technologies due to its simplicity, versatility, and cost-effectiveness in printing intricate and complex tissues (Zhang et al., 2023). DLP bioprinting is a form of bioprinting process based on modified stereolithography. The operating method uses light to selectively harden a bio-ink layer by layer, additively producing a build (Figure 3B). DLP printers cure bioink planes by plane using a digital light projector. The single-layer projection onto the printing plane for photo-curing in DLP helps it to outperform the other bioprinting technologies. In addition, the printing time is constant in one layer, regardless of the complicated design. Consequently, the printer requires a vertically movable stage, greatly simplifying printer control. As of now, the reported SLAbioprinting technology resolution is around 100 µm and printing durations of below 1 h (Gou et al., 2014) while retaining extremely high cell viability (>90%). This great cell survivability and biocompatibility are due to no external shear stress on cells during bioprinting (Derakhshanfar et al., 2018), as opposed to the extrusion bioprinting technique. However, the material-light interaction, such as the physical characteristics of the bio-ink paired with the photocrosslinking process parameters, heavily influences the mechanical features and resolution of the 3D printed structures (Wang et al., 2018). Based on the photoinitiators, UV or visible light sources are widely employed in the DLP bioprinting technology. Two mechanisms-acryloyl-based crosslinking (Yue et al., 2015) and the thiol-ene click reaction (Greene et al., 2017), are routinely employed to establish covalent bonding in bio-inks by photocrosslinking without considerable cytotoxicity of encapsulated cells. As a result, when utilizing hydrogel macromers as bio-inks, the hydrogel should be modified with an alkenyl or acryloyl functional group. Many academia have published various studies using DLP for 3D bioprinting constructions for orthopedics, particularly cartilage tissue, employing various second-phase polymers (s) such as polyethylene glycol diacrylate (PEGDA) (Zhu et al., 2018), hyaluronic methacrylate (HAMA) (Lam et al., 2019), and silk fibroin methacrylate (SFMA) (Tao et al., 2022).

### 2.3 Gelatin as a 3D bioprinting material for orthopedics applications

Gelatin is an intriguing biocompatible protein with a massive spectrum of physical and chemical features. These fantastic characteristics enable the production of high and low-viscosity gelatin-based bio-inks for various applications, including orthopedics. Gelatin improves bio-ink viscosity for extrusion printing methods because of its temperature-gelation phenomenon. At low temperatures, gelation of gelatin occurs and becomes very viscous; therefore, their physical gelation can temporarily stabilize the printed structure post-printing (Tajima et al., 2018; Yang et al., 2023). In contrast, the low viscosity of the bio-ink is necessary for light-based printing processes. The modified form of gelatin, known as gelatin derivatives, is the most common type used for different tissue engineering applications. One typical modification method is the methacrylation of gelatin, which results in gelatin methacrylate (GM) (Claaßen et al., 2018). Currently, the bio-ink formulations based on GM for specific cell type support to engineer or reconstruct functional tissues are ongoing (Wenz et al., 2017; Sun et al., 2018).

HAp, which accounts for approximately 60% of human B.T., and tricalcium phosphate (TCP) support osteogenic differentiation (Calabrese et al., 2016). These materials (HAp and TCP) are used extensively for orthopedic applications, and bioprinting is no exception. Adding these second-phase materials improves gelatin’s viscosity and its derivatives, which is critical for 3D extrusion bioprinting. However, it also produces biocompatible ink that aids orthopedic regeneration. Wenz et al. (2016) demonstrated a pro-osteogenic impact of GM bio-ink containing HAp. Anada et al. (2019) revealed octacalcium phosphate as a pro-osteogenic influence in GM in a comparable study. They further demonstrated greater vascular sprouting in GM hydrogels by lowering biopolymer concentrations.

Gelatin bio-printing would need effective management of its physical characteristics, especially during extrusion bio-printing because of its temperature sensitivity. Its melting point ranges between 30°C and 37°C based on the bloom intensity, concentration, and pH, making it unstable by melting under physiological conditions. Several ways have been investigated to address this constraint, including forming a permanent peptide link between the amino acids to preserve the structure’s stability at physiological temperature and culture media. Photocrosslinking and enzymatic crosslinking are the most often used techniques to stabilize gelatin-based printed constructs. Both systems have their advantages and disadvantages. The photocrosslinking of methacryloyl groups in GM occurs quickly (in seconds) upon exposure to light or UV, providing structural integrity to the printed construct (Pepelanova et al., 2018). In comparison, enzymatic crosslinking is more time-demanding (minutes) than photo-based crosslinking but less hazardous to cells. Because enzymatic approaches do not have uncrosslinked monomers or generate free radicals, which are not cell friendly, the reason for their cytocompatibility; however, both crosslinking methods provide permanent chain networks, thus providing gelatin chains needed stability for bio-printed structure’s mechanical strength. Enzymes are typically added to gelatin to help form peptide bonds between glutamine-carbonyl group residues and lysine-amino groups in the gelatin chain (Irvine et al., 2015; Naharros Molinero et al., 2024). The peptide bonds stabilize the printed construct and provide mechanical integrity. However, a photoinitiator is added to generate the free radical that initiates the crosslinking in the photocrosslinking method. Because of substrate selectivity, enzymatic crosslinking methods have a low prevalence of side effects. The enzymatic method also removes the requirement for specialized equipment as well as additional photosensitive chemicals that may be harmful (Irvine et al., 2015; Asim et al., 2023).

## 3 Chemical structure and rheological properties of gelatin

**FIGURE 4:**
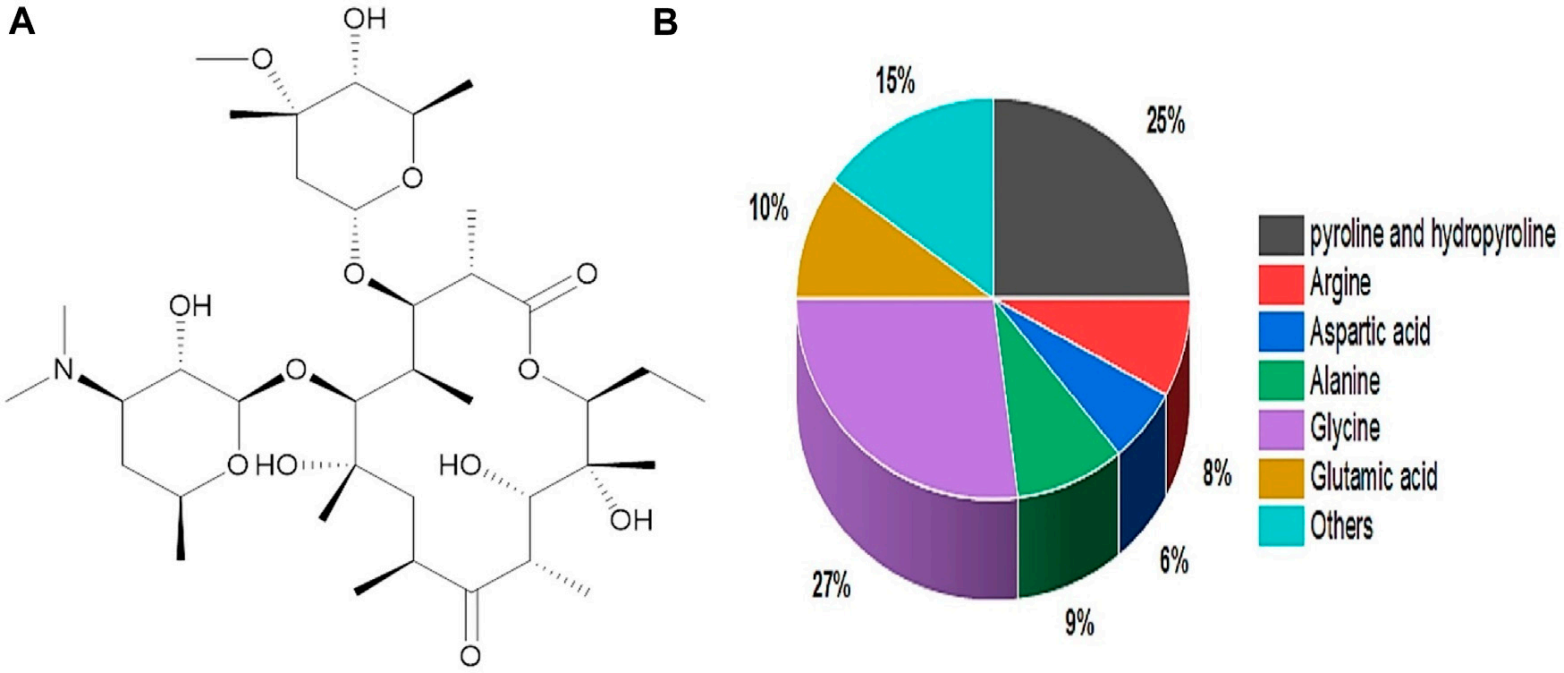
**(A)** Chemical Structure of Gelatin. **(B)** Amino acid percent composition of Gelatin.

Gelatin originated from a Latin word called gelatos, which means frozen/stiff. Gelatin sources include animals via thermal denaturation/partial hydrolysis. Figure 4A depicts gelatin’s chemical structure (Thakur et al., 2017). Gelatin contains around 88% protein, 10% moisture, and 1%–2% salts, with a dry-weight protein concentration of 98%–99% (Valcarcel et al., 2021). In addition, it consists of several molecular weight chains with molecular weights of (240–375 kDa) hydroxyproline, (160–250 kDa) proline, and (80–125 kDa) Glycine. Proline (20%–24%), hydroxyproline (20%–24%), and glycine (27%–35%) are the primary gelatin amino acids (Jafari et al., 2020) (Figure 4B). Gelatin is categorized into two categories based on the pretreatment of gelatin during the extraction process. Type A gelatin with 6–9 isoionic points is derived through an acid treatment process, while type B gelatin with 5 isoionic points is derived via an alkali treatment procedure (Hosseinkhani et al., 2015). In comparison, type A gelatin has a higher important amino acid content than type B, including threonine, cystine, lysine, hydroxyproline, glycine, alanine, proline, isoleucine, and leucine (Alam and Shubhra, 2015). Furthermore, more significant component amounts (concentration) improve gelatin characteristics and boost strength. In terms of bonding, gelatin is stabilized by various covalent bonds, and many weak bonds regulate its flexibility and separation. Gelatin at low temperatures can form hydrogen bonds and give collagen fold shape structure. Furthermore, the hydrogen bonds stabilize the triple helical arrangement, resulting from triple helix glycine residues and developing weak interactions with the oxygen in the carbonyl group (Kessler et al., 2021). Gelatin gel’s rheological or mechanical qualities are essential in characterization and product making, particularly in the pharmaceutical, biomedical, and food industries. It is convenient to make homogenous gelatin gel in a composition between 1% and 50% w/v (Djabourov et al., 1993).

### 3.1 Rheological tunability of gelatin-based printable inks for orthopedic applications

Gelatin has excellent rheological characteristics, but its heat-sensitive nature at physiological temperatures, which significantly impacts these properties, is a disadvantage. Various ways, however, have been used to improve the characteristics or molecular dynamics of gelatin bio-inks. The most common method is to cool it down to achieve a sol-gel transition and print (Bohidar and Jena, 1993). In addition, the addition of biopolymers (Lim et al., 2018; Sathish et al., 2022), proteins (Deng et al., 2021), particles (Diloksumpan et al., 2020; Ratheesh et al., 2020; Tavares et al., 2021), and their mixtures (Benning et al., 2017; Tian et al., 2021) can also improve gelatin printability and shape fidelity. Various researchers have investigated blending gelatin and its derivatives with other polymers to enhance its printability and rheological features. One of these studies combined 5% HAMA and 7% GM to produce a gel with improved rheological characteristics, consistent mechanical qualities, and printability (Lee et al., 2020).

## 4 Gelatin-based hydrogels printed formulation for CTE

### 4.1 Cartilage tissue characteristics

Cartilage is an elastic structural connective tissue with low avascularity and cellularity. Cartilage aids stress absorption, minimizes joint friction, and helps in supporting bone growth. The cartilage tissue’s elasticity features are due to its unique extracellular matrix (ECM), which consists mostly of proteoglycans and collagen II. The cartilage cells secrete a proteoglycan-rich platform, allowing water absorption by the tissue and retaining flexibility. Hydrogel systems resemble cartilage matrix molecular structures. Therefore, hydrogel systems are the best option to repair or regenerate cartilage tissue. In addition, they can supply the necessary biological and physical signals for stem cell and chondrocyte proliferation and differentiation.

The classifications of cartilage include elastic, fibro, and hyaline cartilage (Wang et al., 2022a; Sha ban, 2024). The elastic cartilage has a dense network of collagen elastic fibers responsible for its flexibility. Typical examples of elastic cartilage are the outer ear and epiglottis. On the other hand, hyaline cartilage possesses a closely packed smooth surface collagen network, which helps it be flexible and tough. In addition, hyaline cartilage is the commonest cartilage, and bone joint articular cartilage is a typical example. Furthermore, the cartilage within the bone, which acts as an ossification center or growth plate, is another example of hyaline cartilage. However, fibrocartilage is the best suited for support and stiffness due to bundles of collagen fiber embedded with chondrocytes; hence, it is the toughest among all cartilages. Fibrocartilage is present in the meniscus and intervertebral discs in articulating joints. The mechanical qualities of hyaline cartilage, such as articular cartilage, are caused by its biphasic nature. Water interacts with ions in the fluid phase, whereas collagen fibers interact with proteoglycans in the solid part. The liquid phase shift in the fibrous network under loading results in the tissue’s viscoelastic nature (Wilson et al., 2005). Proteoglycans’ charged sidechains preserve the differential osmotic pressure within the surrounding tissues and cartilage, helping in tissue viscoelastic behavior and water retention and the of the tissue.

Articular cartilage absorbs shock and cushioning in the joint when the body moves. Articular cartilage is extensively dependent on its compressive qualities. As a result, any materials intended for articular cartilage regeneration via the bio-printing method should possess comparable attributes. Furthermore, the intervertebral disc (IVD) structure is more complicated than the articular cartilage structure (Crump et al., 2023; She et al., 2023). The IVD annulus pulposus concentric fibrous layers encircled the nucleus pulposus, which carries the axial compressive loads. The cartilaginous endplates link the intervertebral discs’ top and bottom surfaces to the surrounding vertebrae. The IVD’s primary purpose is to take up and disperse loads applied to the spine when we move. Gravity’s axial compression is the fundamental stress on discs. The outer annulus fibrosus distributes the pressure axially. In addition, the body movement torsion lateral and bending stresses are also taken care of by IVDs. Furthermore, IVDs have compressive moduli ranging from 10 to 20 MPa and tensile moduli ranging from 2.6 to 3.5 MPa. Nevertheless, its mechanical shear stiffness is an essential attribute in our body’s axial movement and also involves the discs, which range from 20 to 300 N/mm anterior-posteriorly and 40–300 N/mm laterally (Grace et al., 2015). The material replicating the nucleus pulposus must be a firm supporting hydrogel. The material imitating the annulus fibrosus must include fiber-reinforcing aligned sections that can withstand axial stresses. When creating material solutions for particular cartilage tissues, tissue architecture, and mechanical qualities must be addressed because gelatin provides a hydrogel system with easily modulable mechanical characteristics based on the crosslinking concentration and degree and, as a result, commonly utilized for cartilage 3D bioprinting. Adding second-phase materials or changing gelatin bio-ink formulations can facilitate tunability to meet the desired cartilage tissue. The typical biomechanical properties of cartilage are illustrated in Table 1.

**TABLE 1:** Example of biomechanical properties of cartilage (Kabir et al., 2021).

Biomechanical properties	Properties value
Poisson ratio	0.4 ± 0.1
instantaneous modulus at 1 mm/s loading rate	52.14 ± 9.47 MPa
Young’s modulus	1.03 ± 0.48 MPa
Equilibrium modulus	7.48 ± 4.42 MPa
Compressive modulus	10.60 ± 3.62 MPa
Dynamic modulus at 1 Hz	7.71 ± 4.62 MPa
Loss factor	0.11 ± 0.02

### 4.2 3D-printed cartilage construct requirements

Cartilage regeneration is a considerable clinical concern because of the tissue’s avascularity and poor cell density. Only symptomatic therapies are available for cartilage defects caused by illnesses such as osteoarthritis or trauma. In addition, autologous, abrasion chondroplasty, and allograft are advanced surgical methods only performed in chronic patients. These procedures, however, have many disadvantages, such as graft necrosis, donor site morbidity, shortage of donor sites, and absence of desirable geometry. Therefore, tissue engineering technologies that can assist cartilage tissue regeneration without requiring invasive surgery are an appropriate therapy option. Gelatin-based biomaterial systems can readily offer biophysical signals for cartilage cell growth and differentiation by mimicking the hydrated state of cartilage tissue. As previously indicated, the material characteristics of synthetic cartilage tissue will be the critical element in developing the bio-ink formulation.

Gelatin and its derivatives have previously been utilized with other materials to satisfy the demands of various cartilage tissues. According to one research, a larger quantity of GM + HAMA increased bio-ink stiffness and the synthesis of cartilaginous proteins matrix, resulting in a high premature phenotype. Even after ECM formation, the resultant biomimetically stratified structures preserved their gradient-like system and significantly increased COL2A1 gene expression (+178%) (Shopperly et al., 2022). Similarly, Deng et al. (2021) found that the combination of GM and SFMA gel showed acceptable mechanical characteristics *in vitro*. However, the combination of GM and SF grafted parathyroid hormone (SF-PTH) gel reduced chondrocyte enlargement and was advantageous in producing hyaline cartilage ECM. The *in vivo* investigations showed that the scaffolds derived from the variety of GM and SF-PTH/GM and SFMA gels enhance osteochondral engineering and retain a large amount of hyaline cartilage phenotype. Hence, combining gelatin with other biopolymers has not only shown outstanding mechanical features but also demonstrated an improved functionalities.

Gelatin and its derivative’s compression modulus may be easily increased by increasing crosslinking density and weight by volume (w/v) composition, although the acceptable shear/tensile characteristics are difficult to acquire. Mixing gelatin with other polymers produces interpenetrating networks with enhanced crosslinking capabilities. The hydrogel can sustain tensile/shear stresses because of its interpenetrating network. Several investigations have looked into gelatin in conjunction with several other methacrylate biopolymers. A classic example is research in which they created a printing resin of 10% GM and varied amounts of PEGDA. They observed that incorporating PEGDA into GM ink considerably increases printing resolution. Furthermore, the compressive investigation reveals that the modulus of the bio-printed scaffolds rises proportionately with the concentrations of PEGDA (Zhu et al., 2018).

### 4.3 Gelatin-based bio-ink formulations for CTE

Several cartilage engineering experiments employed gelatin and its derivatives as the primary inks and bio-inks components. Most of these studies focus on creating novel printable functional materials that might be cell-friendly and enable the printing of cartilage tissue, including ear or some meniscus portion, with the goal of tissue engineering. Printed cell-laden, well-defined constructs with patient-specific geometries have the potential to function as space fillers (Kreller et al., 2021; Murali and Parameswaran, 2024). Gelatin is mixed with other materials to enhance the scaffold’s material printability, mechanical characteristics, and long-term stability. In addition, biological activity (usually MSCs or chondrocytes) includes cell spreading, differentiation, and proliferation. Physical sol-gel transformation and enzymatic, chemical, or photochemical crosslinking are all used crosslinking processes. However, the differentiation of MSCs needs a few weeks of culture for chondrocyte formation; therefore, the long-time stability of cell-encapsulated scaffolds is critical (Chu et al., 2021). Table 2 comprises the recently developed gelatin-based bio-inks and the study parameters.

**TABLE 2:** Examples of gelatin-biopolymers based bio-inks formulations for CTE.

Ink preparation	Crosslinking procedure	Structure printed	Used cells	Bioprinter	Remarks	Ref.
PRP	UV	Disk of cylinder shape	ATDC5	3D bioprinter extruder-based	Better mechanical properties and better release of growth factor	Irmak and Gümüşderelioglu (2020)
SF	Mushroom tyrosinase	Square holes gride	TVA-BMSCs	Robotic stage	Improved mechanical properties, cartilage articular cell differentiation and proliferation	Chawla et al. (2017)
SF	—	Structures of ear and grids	porcine ear primary chondrocytes	Bioprinter microextrusion-based	Improved degradation, mechanical, and cell viability. Chondrogenic gene upregulation	Singh et al. (2019)
SF and Alginate	CaCl_2_ and mushroom tyrosinase	Square mesh	hMSCs	RegenHU Bioprinting platform	No effect on cell viability and improved rheological properties	Trucco et al. (2021)
Alginate, PVA, and HA-PBA (phenylboronic acid grafted hyaluronic acid)	CaCl_2_	Meniscus mesh	ADMSC from rabbit	3D Bioplotter	Showed better printability, anti-oxidant, and cytocompatibility	Shi et al. (2022)
Oxidized alginate	CaCl_2_	Cylindrical mesh	MSCs from pig	Bioprinter RegenHU	Showed better MSCs chondrogenic differentiation	Barceló et al. (2022)
Alginate and CMC	CaCl_2_	Meniscus mesh	MG63	3D printer BioX	Improved cell differentiation and collagen release, also improvement in the physicochemical properties	Sathish et al. (2022)
Alginate d-aldehyde	CaCl_2_	Mesh	hNSCs	3D printer benchtop (Biobot)	Improved cell migration, cell viability, and mechanical properties	Schwarz et al. (2020)
Alginate sulfate	CaCl_2_ and UV	Cylindrical mesh	MSCs	Bioprinting system (Regen HU)	Improved mechanical properties like toughness and elasticity and promote healthy chondrogenesis	Wang et al. (2021a)
Alginate and polycaprolactone (PCL)	CaCl_2_ and UV	Mesh	BMSCs and CCs	3D Discovery bio-plotter	Improvement of production of articular cartilage mesh	Schipani et al. (2020)
succinimidyl glutarate (SG), PEG, N-carboxymethyl chitosan, and AHA	CaCl_2_	Mesh	Chondrocytes, C2C12, NE-4C, NIH/3T3	3D bioprinter Livprint Norm	Improved room temp printability, biocompatibility, and cell viability	Chen et al. (2021)
Fibrinogen and alginate have low viscosity	Enzymatic: thrombin and CaCl_2_	Cartilage	MSCs of human	Developed by authors	Improved the production of TGF-β	Henrionnet et al. (2020)
HAMA	UV	Rabbit thyroid and larynx, mesh	TMSCs	RegenHU 3D Discovery	Improved mechanical properties, cytotoxicity, and degradation rate for chondrogenesis of TMSCs	Lee et al. (2020)
CSM and HAM	UV	Mesh	Pig Knee cartilage Primary porcine chondrocytes	robot TR300	Improved water content and stiffness as well as cellular behavior	Stier et al. (2019)
Agarose	UV	Spheroids	HMSc	RegenHu bioprinter	Improved chondrogenic phenotype and cellular behaviour	De Moor et al. (2020)
HAMA	UV	Disk of core and shells	hADSCs	Handheld extrusion	Improvement of patient-specific cellular attachments	Onofrillo et al. (2018)
HAMA	UV	Disks (circular) having holes	Primary cell Articular cartilage	DLP	Improvement of patient-specific incorporations of cells	Lam et al. (2019)
PVA	UV	Cylindrical	ACPCs and MSCs	Perfactory^®^ 3 Mini	Improvement of structural fidelity, osteogenic and chondrogenic differentiation, and long-term cell survival	Lim et al. (2018)

### 4.4 Cartilage tissue bio-printing

Gelatin has an intriguing viscoelastic property and chondrogenic potential, making it a clear choice of material for cartilage tissue bioprinting. Concentration variations, other biopolymer additions, and additives applications can also readily change gelatin’s rheological characteristics. Even though gelatin and its derivatives have the required physical qualities as biopolymers for printing and cartilage regeneration, they also have binding or bioactive sites for cell signaling and upregulation of chondrogenic pathways. Despite these characteristics, it lacks structural stability and integrity at physiological temperatures. As a result, hybrid systems that can provide stability to the hydrogel system have received much attention in cartilage 3D bioprinting. Therefore, the subsequent paragraph entails a summary of these strategies.

Researchers have used methacrylic groups to create photochemical polymerizations by functionalizing gelatin. The fabrication of physiologically stable and crosslinked structures is achievable by using photoresponsive polymer. In a study (Gu et al., 2018), the encapsulation of primary human chondrocytes in 10% (w/v) GM printed scaffold assisted by a reversible physical crosslinking technique. In addition, the UV light irreversibly crosslinked these printed constructions, ensuring their stability. Encapsulated chondrocyte metabolic activity and proliferation were higher in chondrocytes printed at ideal temperatures than in lower temperatures.

**FIGURE 5:**
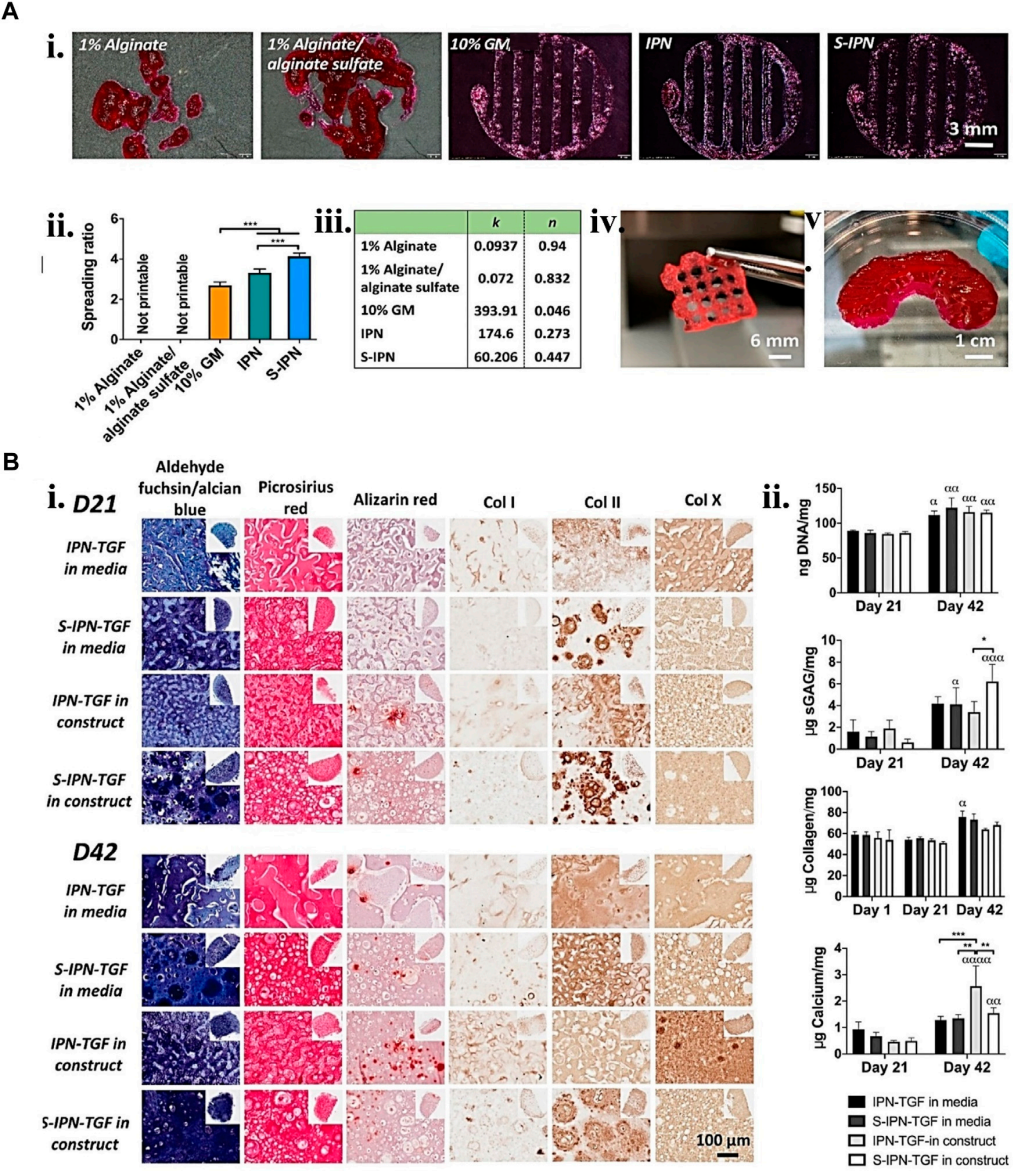
Gelatin biopolymers-based bio-ink for CTE showing **(A)** printability. i. Different gelatin-based formulations 3D printed structure ii. Printed structure spreading ratio. iii. Shear thinning coefficient data from fitting power law to the shear viscosity linear region of the various bio-inks. iv. S-IPN Printed 3D grid construct. v. S-IPN printed meniscus construct. **(B)** S-IPN and IPN *in vitro* chondrogenesis study using TGF- β3 in either construct or cell culture media. i. Immunohistochemical and histological staining for calcium, collagen, sulfated glycosaminoglycan (sGAG), collagen type I, II, and X deposition at day 21 and 42 time points. ii. quantification of collagen, DNA, sGAG, and calcium deposited per construct. Reproduced with permission from Wang et al. (2021a).

On the other hand, Lim et al. (2018) used tyramine and methacryloyl to dual-functionalize gelatin. The *in vivo* observation of implanted chondroprogenitor cells inside the printed hydrogel favors neo-cartilage production. The new hydrogel has a glue characteristic that promotes chondrogenesis and allows for safe lateral incorporation into chondral lesions (Figure 5). Hence, the highlighted studies suggest that gelatin-based methacrylic functionalized gel has minimal toxicity and appreciable printability. In addition to being non-toxic, it also supports chondrogenesis in both *in vitro* and *in vivo* studies.

De Moor et al. (2021) discovered how to modify the phenotypic of spheroid-laden structures by altering the physicochemical parameters of the hydrogel. The phenotypic modification aims to determine the influence of the spheroid maturation level prior to bio-printing on the construct phenotype. Interestingly, the late-stage spheroids printed with a 10 w/v % GM ink produced the best outcomes among the other w/v % studied. Therefore, GM is envisaged to be a suitable material for such applications.

Recently, the production of various biopolymer(s) and gelatin-based hydrogels resulted in fine-tuning their physicochemical and biological features. One of those research involved the development of a novel bio-resin by combining GM, methacrylate poly(vinyl alcohol) (PVA-MA), and a visible light transition metal-based photoinitiator. The developed transitional metal-based system has a high molar absorptivity, which aids the bio-printed scaffolds’ high-resolution 25–50 µm features. The high-resolution cell-laden hydrogel constructions with properly printed complicated and organized architecture demonstrated good cell survival, homogeneous distribution, and functioning (Lim et al., 2018). Likewise, in another work, a cell-laden bio-ink including human adipose-derived mesenchymal stem cells (hADSCs), GM, and HAMA was biopen-produced and maintained in the chondrogenic stimulus for 8 weeks *in vitro*. A thorough investigation revealed that the technique resulted in the creation of human hyaline-like cartilage (Onofrillo et al., 2018). Hence, it can be noted from both findings described in this paragraph that the incorporation of the second-phase materials does not pose any harm to the cells but enhances the physical integrity and stability of the printed constructs.

On the other hand, Stier et al. (Diloksumpan et al., 2020) disconnected the traditional association between polymer content, stiffness, and equilibrium degree of swelling. They also investigated building hydrogels with graded hydrogel compositions using layer-wise printing and following multiple tests of various biopolymer combinations combining GM, HAMA, and chondroitin sulfate, including the degree of methacrylate. The resultant glycosaminoglycan-graded hydrogel was stable for 28 days. Finally, the encapsulated chondrocytes were alive and formed a new matrix. Similarly, Lee et al. (2020) extrusion 3D bio-printed various GM and HAMA ratios for cartilage regeneration. The G7H5 (GM 7% and HAMA 5%) bio-ink formulation was best suited for constructing a more intricate larynx geometry, which included the thyroid cartilage, hyoid bone, cricoid cartilage, cervical trachea, and arytenoid cartilage. This bio-ink additionally offered a suitable milieu for the *in vitro* and *in vivo* chondrogenesis of tonsil-derived MSCs (TMSCs). Hence, the tunability of materials offers enormous benefits in CTE.

The present bio-inks are time-consuming and lack the structural support for a high-shape fidelity scaffold. Overcoming this can reduce the duration required for gel preparation and proper cell dispersion and preserve the predetermined geometry during printing with no extra help. Furthermore, high permeability may allow cell proliferation uniformity in bio-printed constructs, thus helping heal homogenous tissue. As a result, a good permeability time-sharing structure-supporting (TSHSP) hydrogel containing 0.75% CMC (carboxymethyl cellulose), 1% AHA (aldehyde-hyaluronic acid), 0.5% 4-arm poly (ethylene glycol) succinimidyl glutarate (PEG-SG), and 1% gelatin was developed in a study. The quick crosslinking dynamic of AHA/N-carboxymethyl chitosan constituted the basis for the TSHSP mechanism. The *in vitro* studies of nerves, muscles, and cartilage cells displayed homogenous cell development and impressive biological specificities (Chen et al., 2021). Including growth factors inside the bio-ink is a potential technique to accelerate tissue regeneration. Heparin’s binding solid affinity for alginate sulfate is a tool that can facilitate its adherence to alginate. This property has been used in one study to create a sulfated interpenetrating network (IPN) bio-ink comprising an alginate sulfate functionalized alginate-GM. This bio-ink was 3D printed and not only allowed the continuous discharge of transforming growth factor-3 (TGF-3) and the release of other proteins. It also creates an environment that helps strong *in vitro* chondrogenesis with no indication of hypertrophy or mineralization over long culture durations (Wang B. et al., 2021). Therefore, a second-phase functional polymer can facilitate and improve gelatin-based bio-ink for cartilage regeneration.

The subjection of printed hydrogel to high quantities of reactive oxygen species (ROS) at defects may impair their phenotypic and functioning, reducing regeneration efficiency. Therefore, an anti-oxidative multifunctional bio-ink is developed in a study to circumvent the ROS challenges. The bio-printed construct increased cell adherence and chondrogenesis of incorporated stem cells. Most notably, after incubation with H_2_O_2_, the hydrogel could protect the incorporated stem cells against overexpression of the MMP13 catabolic gene and ROS-facilitated cartilage-specific downregulation such as ACAN and COL2 anabolic genes (Shi et al., 2022). Hence, small molecules with specific functionalities can be incorporated into a bio-ink formulation for a specified function. This is one of the tremendous benefits that hydrogels offer, especially gelatin-based, which has enormous side chains with the potential for functionalization.

Aside from combining a polysaccharide with gelatin and its derivatives to create a cell-laden architecture, some research has looked at diverse protein-derived biopolymers. Cellular hypertrophy is one of the primary issues with today’s gold-standard synthetic cartilage. This results in temporary cartilage that eventually undergoes endochondral ossification to generate bone trabeculae. Chawla et al. (2017) looked at six 3D bio-printed silk-gelatin scaffold conditions to see which produced the most significant results regarding articular cartilage development. In the presence of TGF-1, bone marrow mesenchymal stem cells (BMSCs) undergo hypertrophic differentiation, whereas in the absence of TGF-1, the incorporated BMSCs in bio-printed silk-gelatin gel undergo articular cartilage-type differentiation.

The majority of biopolymers utilized for cartilage regeneration require crosslinking. As a result, different cross-linkers are rarely employed and are invariably harmful to cells. Therefore, the silk fibroin (SF) capacity to undergo secondary structure formation, which induced gelation coupled with a bulking agent, gelatin, produced a crosslinker-free bio-printed construct. Furthermore, the design promotes encapsulated chondrocyte development and proliferation, as well as the production of cartilaginous ECM. The chondrogenic gene expression increase with limited chondrocyte hypertrophy also confirmed the suitability of the developed formulation (Singh et al., 2019). Another interesting, person-specific polymer source is platelet-rich plasma (PRP), widely used as a therapeutic adjuvant for cartilage injury repair. However, PRP treatments in clinics are unsatisfactory and need improvement, particularly in bioactivity maintenance. As a result, Irmak and Gümüşderelioglu (2020) demonstrated a 3D bio-printed photo-crosslinked cell-laden construct containing GM and PRP for tissue-specific constructions. Analyses of *in vitro* studies indicated an enhancement of ATDC5 differentiation and proliferation in the regularly light-applied GM/PRP gel in the absence of any external chemical agents. More recent examples of gelatin-biopolymers used in bioink for CTE are listed in Table 2.

**FIGURE 6:**
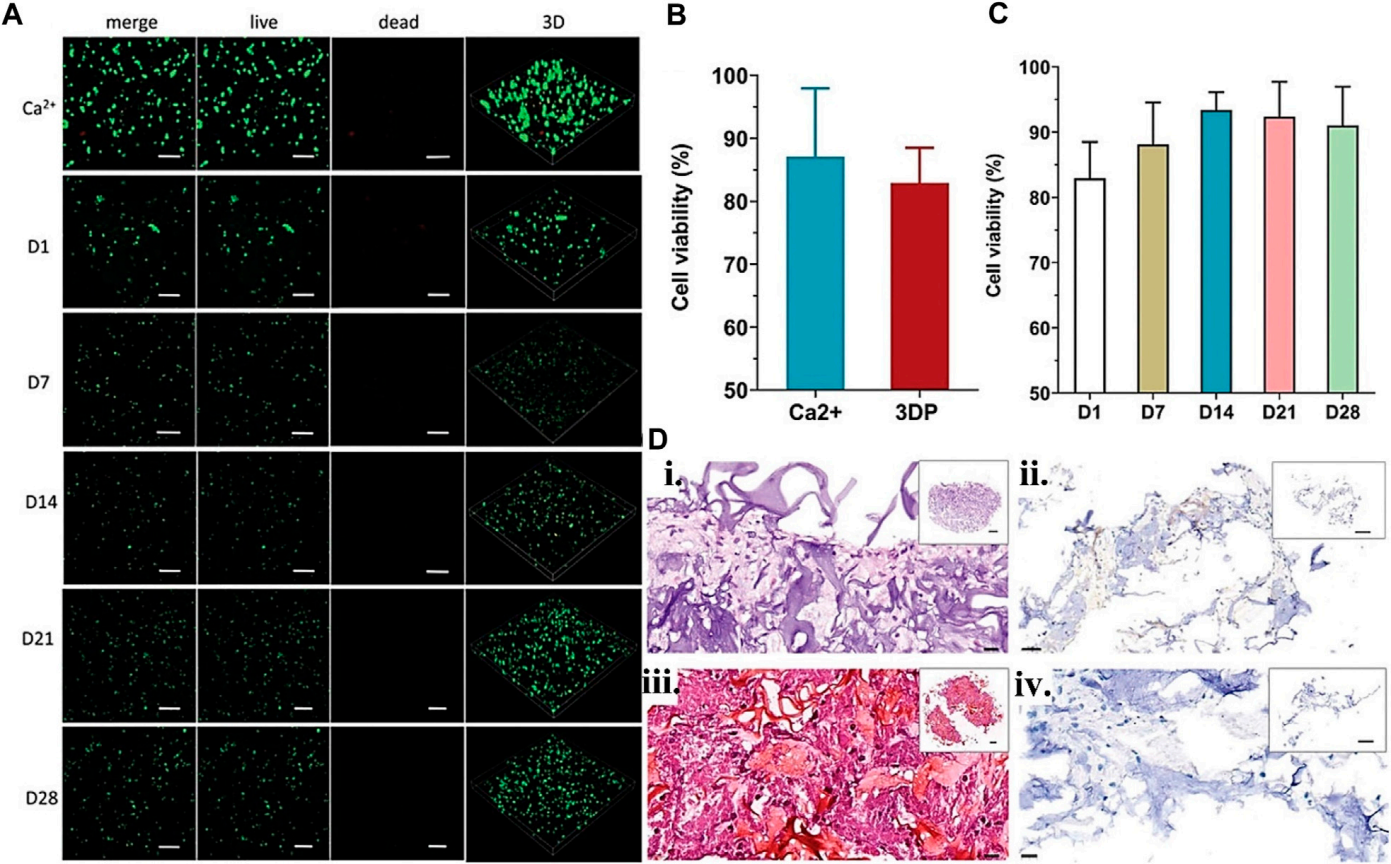
Gelatin plus nanoparticle for CTE showing **(A)** Live/deal cell staining confocal image for BMSCc encapsulated scaffolds from day 1–28-day point with no significant difference in viability. **(B)** The Ca^2+^ physically crosslinked and 3D-printed BMSCs viability at day 1 using unpaired t-test, and **(C)** for various day points using one-way ANOVA. **(D)** *In vitro*, scaffold cartilage paraffin sections stained with (i) H&E, (ii) immunohistochemistry, and Safranin O/Fast Green for (iii) type II collagen and (iv) aggrecan. Reproduced with permission from Wang et al. (2022b).

Mixing gelatin and its derivatives with particulate materials may improve its bioactivity and mechanical characteristics. Kosik-Kozioł et al. (2019) proposed a biomimetic bio-printed gel comprising β-tricalcium phosphate (TCP), alginate, and GM for creating a calcified type of cartilage using an extrusion-based bioprinting approach. The printed structures suitability assessment for cartilage regeneration using RT-qPCR for gene expression quantification such as osteogenic (ALPL, BGLAP) and important chondrogenic (COL1, COL2, COL10A1, ACAN) gene markers. Additionally, fluorescent immunocytochemistry assesses the printed construct quality. Another study used three different bioprinting techniques to bioprint GM, calcium phosphate, and glycosaminoglycan additive biopolymer to assess which bioprinting technique is more cell-friendly. Among the three examined 3D bioprinting processes, DLP printed structures permitted the most significant observed growth in cell number after 7 days (Bedell et al., 2022). The results demonstrate how different bioprinting methods can affect the viability of the cell-laden construct. In another study, a short fiber-reinforced double-network bio-ink was 3D printed to provide an anatomically correct and mechanically adjustable construct for CTE (Figure 6). The addition of short PLLA fibers increases printing fidelity and promotes the generation of mechanically robust constructions. Furthermore, this mechanically reinforced alginate/GM double-network bio-ink is biocompatible and promotes *in vitro* chondrogenesis of bone marrow-derived stromal cells (Wang et al., 2022b). Hence, particle materials have been shown to provide bioactivities and mechanical functionalities to printed constructs. More recent examples of gelatin particle-based use in bioink for CTE are listed in Table 3.

**TABLE 3:** Examples of gelatin-particles-based bio-inks formulations for CTE.

Ink preparation	Crosslinking procedure	Structure printed	Used cells	Bioprinter	Remarks	Ref.
Methacrylated alginate and short fiber of PLLA	CaCl_2_	Cylindrical	BMSCs human	BIO X 3D bioprinter	Improved mechanical properties by adjustment of fibre aspect ratio and improved *in vitro* chondrogenic induction	Wang et al. (2022b)
HAp and alginate	CaCl_2_, UV	Disk shape	Male rats BMSCs	Bioprinter (EFL-BP-6601)	Improved mechanical properties and chondrogenic differentiation	Zhou et al. (2021)
β-TCP, NFC, xanthan gum (XG), HAMA	UV	Crosshatch type structure	hMSCs human	3D Discovery bioprinter	Improved cell viability and gel stability	Bedell et al. (2022)
CNC and HAMA	UV	Mesh	ATDC5	Bio-plotter	Improved mechanical properties and cell viability	Fan et al. (2020)
β-TCP and Alginate	CaCl_2_ and UV	Mesh and square holes	BM-hMSCs	3D-Bioplotter	Enhanced mechanical and biological properties	Kosik-Kozioł et al. (2019)
Glycerol and fibrinogen, HAp	Enzyme Thrombin	Cylindrical-like mesh structure	BMSC	Organ printing united system bioprinter	Enhanced cartilage repair effect	Sun et al. (2021a)
C-PCaP (cross-linkable PCaP), methacryloyl, NC-PCaP (non-cross-linkable PCaP), PCaP (printable calcium phosphate) and P-CL-MA (poloxamer grafted caprolactone oligomers and methacryloyl)	TEMED (tetramethylethylenediamine)/APS(ammonium persulphate)	Circular structure	ACPCs	Electrowriting device	Allowed cartilage and bone regeneration	Diloksumpan et al. (2020)

**FIGURE 7:**
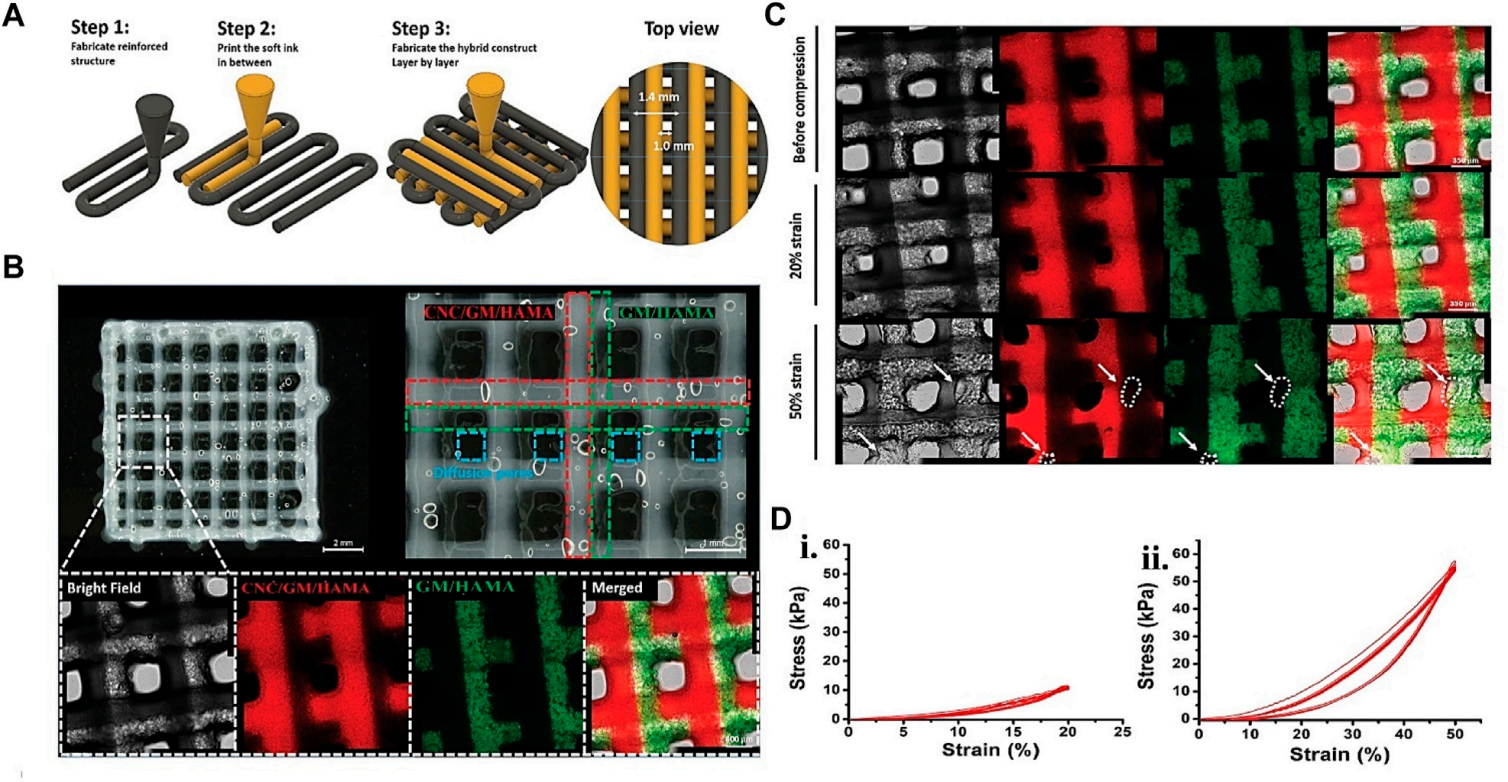
Gelatin plus polymer for CTE showing **(A)** Schematic representation of hybrid printing steps and **(B)** Hybrid printed confocal and optical micrographs of the hybrid printed scaffold. The confocal image’s green and red dotted lines represent the GM/HAMA and CNC-reinforced biopolymer, respectively. The red fluorescence signifies the rhodamine-labeled GM for GM/HAMA, while the green part is the FITC-labeled GM for the CNC-reinforced system. **(C)** Confocal images showing the hybrid structure integrity after 20% strain compression in 10 cycles. The white dots and the arrow on 50% strain compression analysis result in 10 cycles revealing structural defects. **(D)** (i) stress-strain graph for 20% strain during 10 compression cycles. (ii) stress-strain graph for 50% during 10 compression cycles. Adjusted and reproduced with permission from Fan et al. (2020).

PCL and other synthetic polymers have previously been used to support 3D printing cell-laden structures for self-standing tissue constructions. These strategies are frequently implemented concurrently (i.e., simultaneous printing of plastics and cell-laden bio-inks). Research proved that auricular cartilage repair employing PCL as scaffolding, GM and HAMA layers printed in between, and Lutrol F-127 as sacrificial material. The obtained mechanical characteristics of the resultant hybrid construct are like those of natural cartilage. The printing procedure did not affect the proliferation or viability of hMSC supplied inside the bio-ink (Chung et al., 2020). In another work, PCL microchambers were pre-printed to direct the formation of cellular spheroids. The biomechanical characteristics and composition of the bio-printed construct were comparable to natural cartilage (Daly and Kelly, 2019). In the same version, Ruiz-Cantu et al. (2020) bio-printed chondrocyte cell-laden GM in between PCL plastic structures to improve the mechanical and biological qualities of the developed 3D structure. After 50 days of culture, the 3D bio-printed constructions revealed neocartilage development and mechanical rates similar to nasal alar cartilage. The composite structures’ collagen type II and glycosaminoglycans presence also demonstrated neocartilage development. Even though PCL provides hydrogels with considerable mechanical support, its structural integration with hydrogel is problematic. To find better-automated support solutions. In one study, cellulose nanocrystals support the structural component of a mechanically reinforced hydrogel ink and GM/HAMA ATDC5 cell-laden bio-ink (Figure 7). The printed hybrid scaffolds displayed high mechanical stability, and the printing phase did not primarily affect the survival of ATDC5-encapsulated cells in the scaffold (Fan et al., 2020). More recent examples of gelatin hybrid system use in bioink for CTE are listed in Table 4.

**TABLE 4:** Examples of Gelatin-hybrid-based bio-inks formulations for CTE.

Ink preparation	Crosslinking procedure	Structure printed	Used cells	Bioprinter	Remarks	Ref.
PCL	UV	Mesh	Sheep Chondrocytes	REGENHU 3D	ECM secretion and cell proliferation improved	Ruiz-Cantu et al. (2020)
Pluronic PCL and HAMA	UV	Cartilage and Mesh	MSCs	3D Bioplotter	Cellular behavior of MSCs not affected	Chung et al. (2020)
PCL	UV	Mesh Cartilage Structure	AuCPCs	Multinozzle bioprinter	Ear structure showed excellent shape fidelity, cell viability, and cartilage matrix deposition	Otto et al. (2021)
Pluronic sacrificial ink with PCL	UV	Micro channels	Chondrocytes and BMSCs	Inkjet	Excellent ear (human) construct and Development of cell spheroidals	Daly and Kelly (2019)

## 5 Gelatin-based hydrogels printed formulation for BTE

### 5.1 Natural bone characteristics

Bone gives the human body structure and stability. It is a mineralized hard tissue that may regenerate on a smaller scale. More minor bone fractures heal on their own, and they are also often treated using the clinical casting method. However, in case of a significant fracture defect, the defect site will not heal without the help of implants or surgical intervention. Significant bone fracture defects, also called critical-sized bone defects, are often 1–2 cm in size or bigger or when the bone circumference loss is >50% owing to disease, high-energy trauma, or accident (Nauth et al., 2018; Liu F. et al., 2023; Han et al., 2023; Su et al., 2023; Xia et al., 2023). These deformities are challenging to treat since surgical procedures only stabilize the bone fracture. Still, biological material is required to occupy the defective space and assist in new tissue formation. As a result, bioprinting technology, coupled with tissue engineering, offers enormous promise for treating large bone defects. Additionally, printing necessary shapes using clinical defect area photographs to achieve a correct match. There are two kinds of bone tissue: cancellous and cortical. The cortical bone is a thick, dense exterior layer that accounts for 4/5 of bone mass. Cortical bone comprises densely packed osteons with concentric rings surrounding a central canal. Additionally, cortical bone is anisotropic and has a 5%–15% porosity (Morgan et al., 2018; Rodriguez Palomo et al., 2023; Zhou et al., 2023). Furthermore, the cortical bone has a transverse elastic modulus of 10.1 ± 2.4 GPa and a longitudinal elastic modulus of 17.9 ± 3.9 GPa (Reilly and Burstein, 1975). The less dense, lighter, and spongy interior is called cancellous bone and comprises trabeculae. To produce the core bone, the cancellous bone forms a thin but robust interconnectivity with a porosity of about 40%–95% (Morgan et al., 2018). Finally, trabecular bone moduli vary from 10 to 3,000 MPa (Morgan and Keaveny, 2001; Siriphannon and Rukchonlatee, 2023).

The bio-ink bone ultrastructure imitation can boost the production of mechanically robust bone in an *in vivo* environment since the mechanical qualities of bone are challenging to recreate in the bio-printed strategy. Bone is a nanocomposite containing the protein-mineral crystal. The ECM of bone contains collagen type I, which functions as a scaffold for crystallizing the deposited calcium phosphate to carbonated HAp nanocrystals (Murab et al., 2020). Combining collagen type I present in amino acid residues of hydroxyproline with HAp produces relatively large binding energy nanocomposites between 63 and 126 kJmol^−1^ (Cutini et al., 2019). Because of this robust nanocomposite ultrastructure, bone tissue has high compressive strength and flexibility. Therefore, if the bio-inks formulation has a similar chemistry to HAp nanocomposite production, in that case, they may be able to aid in the regeneration of mechanically robust bone tissue. Thus, gelatin-based biopolymer has the potential to be a chemically acceptable bio-ink solution for bone tissue bioprinting due to its inherent cell-friendly behavior and the possibility of its mechanical structure tunability.

### 5.2 3D printed bone constructs requirements

The present surgical procedures for treating large-size bone deformities involve induced membranes, allografts, autografts, and transfer (bone). However, all the mentioned surgical procedures have drawbacks (Bezstarosti et al., 2021; Dalfino et al., 2023). Autografts have the flaws of lack of geometry conformity, secondary morbidity, and a lack of greater graft availability. Conversely, allografts have disadvantages such as transfection, core necrosis, and lack of geometry conformance. Until recently, tissue engineering concepts have served as excellent solutions to all the problems associated with the current therapy methodology. Shape conformation is solved via 3D printing and bioprinting since scaffolds are fabricated in the precise shape and size of the defect location utilizing the defect area medical pictures. The primary critical requirement of the biopolymer is to give recruited cells with a bone-like ultrastructure and chemistry. This bone-like chemistry would provide progenitor cells like BMSCs with physical and chemical signals to develop toward osteogenesis and bone-unique ECM deposition.

Various mineral particles such as HAp may be mixed with hydrogels such as gelatin to improve the bio-ink mechanical qualities such as stiffness while also giving biochemical signals for bMSC development. In one study, after 14 days of printing, a biopolymer system involving HAp, gelatin, and alginate stimulates the osteogenic differentiation of (adipose-derived mesenchymal stem cells) ADSCs (Wang et al., 2016). In contrast, in the control group (alginate-gelatin), the bio-ink system containing HAp improved bone deposition in the mice. The improvement in bone mineralization suggests that particulate HAp can activate osteogenic signaling pathways, creating mineralized, mechanically stable bone tissue. In another study, Sharma et al. (2019) recently bio-printed SF-G-CaCl2 constructs to investigate the effect of calcium release in bone formation. However, the bio-printed scaffolds containing calcium particles enhance the osteogenesis of hMSCs via 1) upregulating the osteogenic markers like OPN, RUNX2, ON, alkaline phosphatase (ALP), and COL I gene expression, 2) upregulating the osteocytic markers such as PDPN, SOST, and DMP 1 gene expression; 3) upregulating the BMP2, BMP4, and β-catenin gene expression; and 4) facilitating mineral deposition, then the scaffolds without calcium. As a result, biochemical and biophysical signals can facilitate the development of gelatin-based bio-inks for BTE. Although, composite formations of mechanical toughness offer a better significant benefit since they can maintain the bio-printed scaffolds *in vivo* while aiding in more bone formation.

### 5.3 Gelatin-based bio-ink formulations for BTE

Based on the literature, the wide use of gelatin-based biopolymers as materials for printing bone regeneration scaffolds is apparent. The investigations generally focused on techniques to improve the material’s osteogenic differentiation capability and mechanical characteristics of the scaffolds, as well as on achieving increased vascularized structures (Alcala-Orozco et al., 2020; Zhang et al., 2020; Yang et al., 2022). Pure gelatin or its derivative inks and mixes with other biopolymers, similar to cartilage printing inks, were proposed. Furthermore, printed materials loaded with bioactive materials, including calcium oxide or silica nanoparticles (Tavares et al., 2021), active glasses (Ojansivu et al., 2019), HAp nanoparticles (Allen et al., 2022), or tricalcium phosphate (Jalandhra et al., 2022), have been created. The mineral improves the printed scaffolds’ bioactivity, often enhancing mechanical characteristics, biocompatibility, and higher natural tissue biomimicry. The use of photochemical crosslinking is more than other crosslinking types, such as physical and enzymatic crosslinking. Table 3 shows some recent instances of gelatin-based inks for printing in BTE.

### 5.4 Bone tissue bio-printing

Autografts and allografts are still the conventional surgical therapy for large-sized bone defects. Both provide mechanical stability and strong integration but possess several drawbacks. The major weaknesses of autografts remain donor site scarcity and morbidity for significant deformity and the unavailability of preferred shapes for implant fitting. In addition, allografts are costly, rare, and include the danger of disease transmission. Larger allografts acquire necrotic cores because they are not coupled to the host’s circulatory network and generate secondary problems. Therefore, bio-inks with biophysical and biochemical signals that can be bio-printed to match the large defect area might be a feasible technique for such BTE. Irmak et al. (2019) used a microwave approach to create a more elastic and robust 3D bio-printed GM than the standard method. Superior mechanical characteristics, increasing cellular survival, adhesion, proliferation, mineralization, ALP activity, and osteogenic genes mRNA expression levels of preosteoblastic MC3T3-E1 cells were found in the formulated hydrogels. This research exemplifies how gelatin may be adjusted to increase its mechanical properties. Celikkin et al. (2022) 3D printed a 5% GM containing MSC. The *in vivo* findings indicate excellent tissue integration, with no evidence of fibrotic encapsulation or impaired bone growth. Epithelial-mesenchymal interaction (EMI) is an essential element in bone healing. Anything that increases EMI production will inevitably encourage BTE. Recently, epithelial, MSC, and GM cells were 3D printed to aid in promoting EMI by cell recombination. The dimensional culture pattern offered an excellent atmosphere for DPCs and HERS cells to develop mineral deposition patterns, as seen by eosin staining, hematoxylin staining, Masson staining, and immunohistochemistry investigation of the printed construct *in vivo*. As a result of their interactions, they enhance alveolar bone repair (Tang et al., 2022). Hence, bioinks having both physical and biochemical cues offer enormous potential in BTE.

Combining gelatin and its derivatives with other biopolymers to create an interconnected network and a more mechanically stable hydrogel is an effective strategy for increasing bioactivity. During the osteogenic development of MSC, silk-gelatin bio-ink was bio-printed to stimulate the Indian hedgehog (IHH) and canonical Wnt/-catenin pathways (TVA-BMSC). The encapsulated cell’s early differentiation markers, mid and mid-to-late-stage markers, and terminal osteocytic gene expression demonstrate the construct’s suitability. Furthermore, T3 incorporation and endochondral ossification modeling facilitate the activation of Wnt/-catenin, PTH, and IHH pathways. As a result, stem cell osteogenic differentiation potential and mineralization are enhanced (Chawla et al., 2018). In another dimension, Ma et al. (2017) studied the ECM stem cell interactions via a bioprinting approach to achieve an optimal ECM for alveolar bone repair. The bio-ink formulation consisting of PEGDA and GM was bio-printed with periodontal ligament stem cells (PDLSCs). Finally, an *in vivo* investigation employing 4/1 GM/PEGDA revealed that PDLSC-laden gel with an optimal formulation outperformed the other formulations for bone development. Similarly, in another study, three biocompatible biopolymers, HA, hydroxyethyl acrylate (HEA), and GM, were employed as cell carriers for bone cell loading in lattice shapes. The 3D bio-printed product demonstrated stable rheology and outstanding biocompatibility (Noh et al., 2019). These studies revealed the importance of bio-ink formulation to encapsulate cell functionalities. More recent examples of gelatin-biopolymer systems used in bioink for BTE are listed in Table 5.

**TABLE 5:** Examples of Gelatin-biopolymers-based bio-inks formulations for BTE.

Ink preparation	Crosslinking procedure	Structure printed	Used cells	Bioprinter	Remarks	Ref.
GGMA (gellan gum methacrylate)	UV	Disk Mesh	BMSCs and Human Umbilical Vein Endothelial Cells (HUVECs)	3D printer pneumatic extrusion-based	*In vitro* Improved cell viability Angiogenesis and osteogenic potential. Rat model cranial bone defect site improved mineralization and angiogenesis	Li et al. (2022a)
(PEGA8) and (OMA) methacrylated alginate	UV	Bar structure	HeLa, NIH3T3 and hMSC	3D printer (Printrbot)	Improved cell viability and mechanical properties	Ding et al. (2022)
Type I collagen, alginate, and SFMA	Low temp 4°C and UV	Spherical	BMSCs	3D bioprinter PAM-II	Improved cell viability, degradation rate, and compression properties	Chai et al. (2021)
OligoPolyethelene Glycol (OPF) and PEGDA	Low temp 4°C and UV	Square Mesh	MC3T3-E1	BioX 3D bioprinter	Excellent cell viability, proliferation, and better printability	Liu et al. (2021)
Placenta dermis of mouse and alginate	CaCl_2_	Circular mesh	MSC from mice	3D Bioprinter (Regenovo)	Improved sweat gland cells proliferation, migration, and differentiation	Cheng et al. (2019b)
PVA-MA (Polyvinyl alcohol methacrylate)	UV	Matt woven lattice	ECFCs and MSCs	DLP Perfactory^®^ 3 Mini	Improved osteogenic and chondrogenic differentiation	Lim et al. (2018)
Hydroxyethyl acrylate (HEA) and hyaluronic acid (HA)	Potassium peroxodisulfate	Square mesh	MC3T3	3D bioprinting system	Excellent rheological properties, printability, biocompatibility, and cell viability	Noh et al. (2019)
Alginate	CaCl_2_	Rod-model having a lattice structure	hMSCs	Extrusion-based 3D multi-nozzle bioprinter	Soft scaffolds showed excellent mineralizations, cell viability, improved osteogenic differentiation	Zhang et al. (2020)
PEGDA	UV	microarray	PDLSCs	Pressure extrusion bioprinter	Improved cell differentiation and proliferation, cell viability	Ma et al. (2017)
Hydroxyl apatite and HAMA	UV	Square network	hASCs	top robot TR300	Improved remodelling and production of the bone matrix and improved cell viability	Wenz et al. (2017)
HAp, Alginate, Fibrin, Collagen, and Metrigel	CaCl_2_ and thrombin	--	Mononuclear cells (MNCs)	DoD dispenser piezo-driven	No negative effect was seen on the biocompatibility and cell viability of MNCs	Benning et al. (2017)
SFMA And SF grafted Parathyroid hormone	UV	Circular mesh	BMSCs and ACs	3D bio-printer (Regenovo)	Improved mechanical properties and cell viability	Deng et al. (2021)
HA, glycerol, HA, and fibrin	Low temp 4^o^C and thrombin	Cube	ASC and MSCs	syringe pump piston-driven	Outstanding mechanical properties, cell viability, biocompatibility, and osteogenic differentiation	Wehrle et al. (2019)
SFMA	UV	Square network	BMSCs and HUVECs	3D bioprinter PAM-II	Rat bone cranial defect showed osteogenic potential and angiogenesis	Yang et al. (2022)
SF	Mushroom tyrosinase	Holes and mesh-like grids	TVA-BMSCs	Robotic stage	Improved osteogenic gene expression and mechanical properties	Chawla et al. (2018)

**FIGURE 8:**
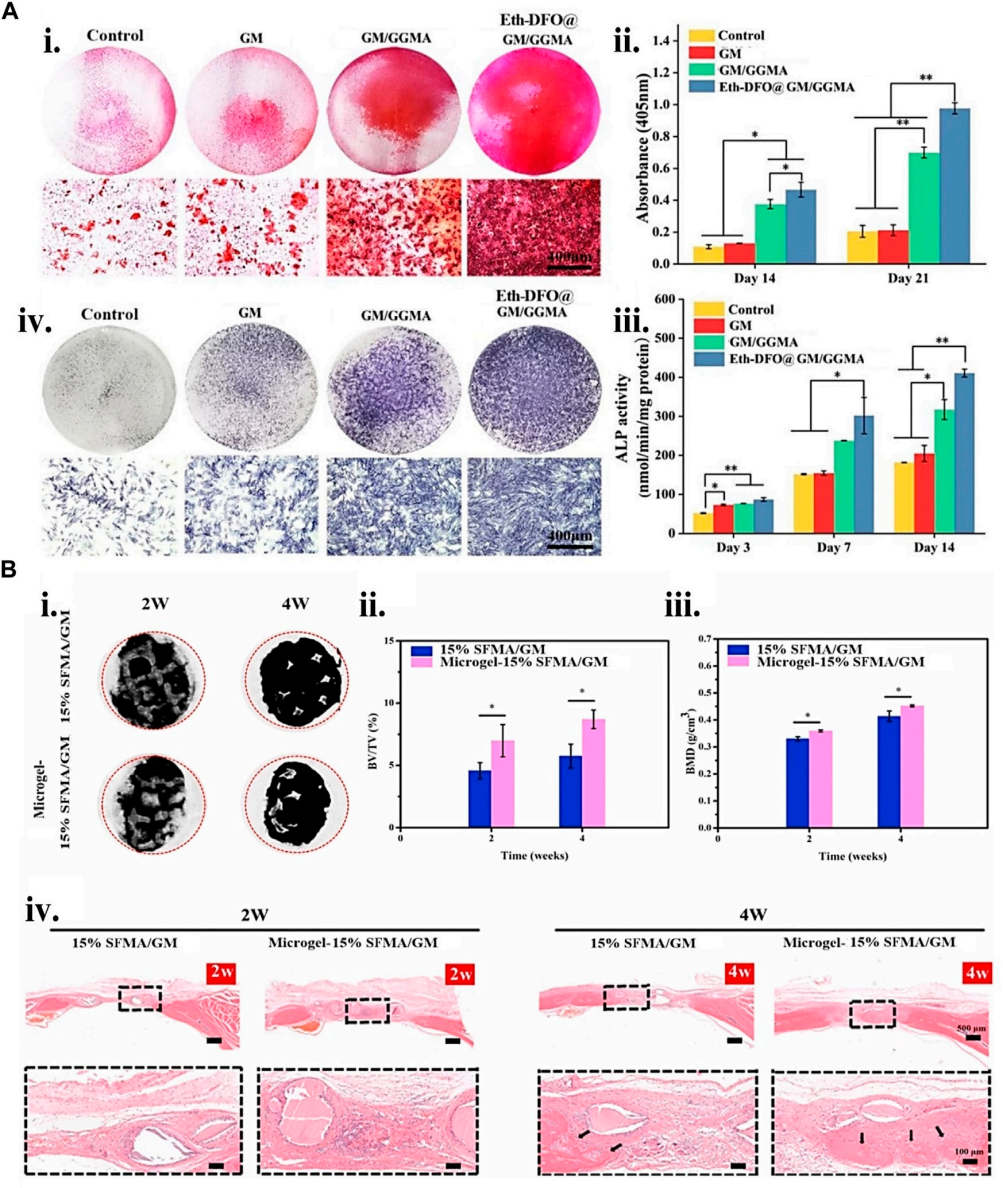
Gelatin biopolymers-based bio-ink for BTE showing **(A)** deferoxamine (DFO)-loaded ethosomes GM/GGMA (Eth-DFO@GM/GGMA) construct osteogenic and angiogenic capacity *in vitro*. (i) HUVECs revealing Analytical and original tube formation post 9 h culture. (ii) the number of meshes, master segments, and the number of junctions in quantitative analysis. (iii) 21 days BMSCs culture of Alizarin Red S (ARS) staining digital and microscopic images of various bio-ink groups. (iv) Day 14 ALP staining of BMSCs encapsulated construct microscopic and digital images. (v) ALP activity quantitative analysis. **(B)** (i) Micro-CT micrograph of 2 and 4 weeks post-implantation of 3D reconstruction in a rat cranial bone defects. (ii) 2 and 4 weeks bone volume (BV)/tissue volume (TV) and (iii) bone mass density (BMD) analysis postoperatively. (iv) H&E staining histological analysis at 2 and 4 weeks. The black arrows indicate the new bone formation. Adjusted and reproduced with permission from Li et al. (2022a).

To protect cells against extrusion printing shear stress, the use of microgel cell encapsulated core-shell structure is desirable. One study (Fan et al., 2020) fabricated a core-shell structure consisting of an alginate shell layer and type I collagen core layer microgel using a multichannel microfluidic device to achieve a better cell viable product. The materials SFMA, GM, and microgels were combined and 3D printed. Compared to a 15% SFMA/GM construct, the microgels-15% SFMA/GM construct demonstrated improved biocompatibility and bone formation capability. In most fabrication processes, including additive manufacturing, quickly developing efficient vascularized tissue by 3D-printed constructs remains difficult. Li et al. (2022a) presented a solution to this problem whereby they established and bio-printed a new bio-ink formulation consisting of deferoxamine (DFO)-loaded ethosomes (Eth), GM, and GGMA. The sustained release of DFO from the gel having DFO enhances its mineralization, migration of endothelial cell and tube formation, and osteoblast ALP expression (Figure 8).

**FIGURE 9:**
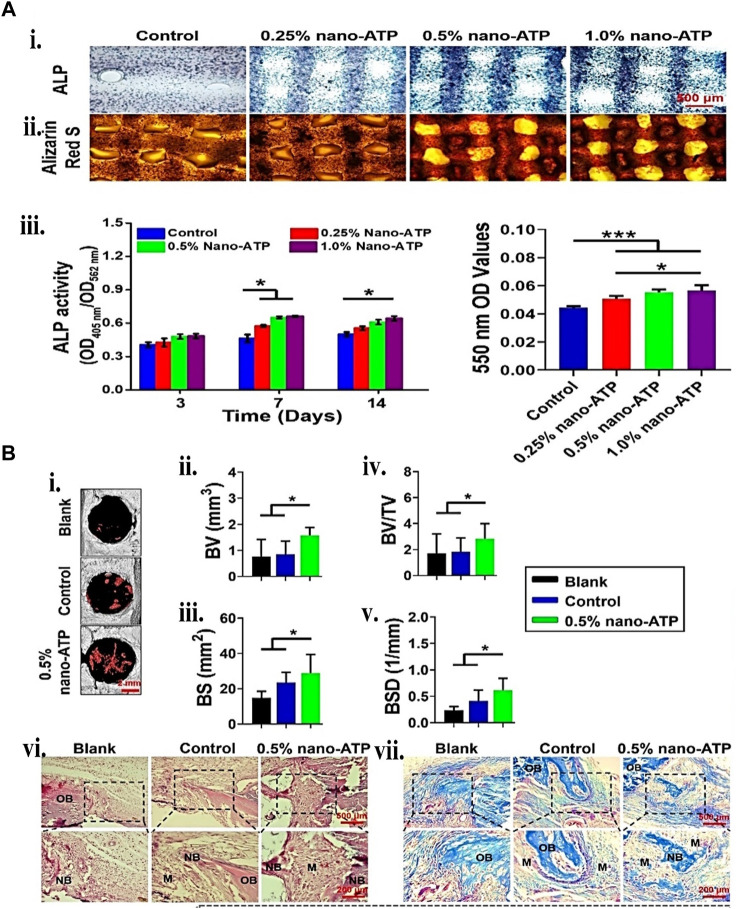
Gelatin plus nanoparticle for BTE showing **(A)** 3D bio-printed BMSCs-laden cell-instructive osteogenesis ability. (i) Day 14 ALP and ARS staining micrograph. (ii) ALP quantitative activity measured between the 405 nm and 562 nm ratio. (iii) calcium minerals deposition quantification by measuring the ARS mineralized stained in the scaffolds. **(B)** New bone formation histological and micro-computed tomography (μCT) assessment. (i) 2 weeks post-surgery, 3D reconstructed μCT micrographs. The red mark inside the circle indicates the new bone formed. (ii) BV, (iii) bone surface (BS), (iv) BV/TV, and (v) bone surface density (vi) 2 weeks bone defect H&E and Golder’s Trichome staining. (vii) Day 5 and 7 Osteoclasts TRAP staining. Reproduced with permission from Liu et al. (2023b).

Because gelatin is short of physical stability, adding nano or microparticles may improve the bio-ink physical and mechanical characteristics through nano/micro composites. Interestingly, using the same particle may additionally aid regeneration by carrying bioactive compounds. These mechanical characteristics increases may give biomechanical signals for differentiating mesenchymal stem cells into osteogenic lineages. One recent study (Chung et al., 2020) showed that Laponite^®^ (LPN) and GM were 3D bio-printed to create effective cell-instructive scaffolds. The *in vitro* nanocomposite study demonstrated high form integrity, human bone marrow mesenchymal stem cells (hBMSC) survivability, and improved osteogenic differentiation support. VEGF-loaded LPN-GM scaffolds revealed considerably greater vascular penetration than GM-VEGF scaffolds. Yu et al. (2020a), on the other hand, bio-printed two bio-polymer gels of strontium-doped calcium silicate powder (FGSr) and fish gelatin methacrylate (FGM). The bio-printed composite outperformed FGM scaffolds regarding mechanical properties, biocompatibility, and osteogenesis differentiation of human Wharton jelly-derived mesenchymal stem cells (WJMSC). Similar results were obtained by embedding silanated silica particles (Choi et al., 2021) and nano-attapulgite (nano-ATP) (Figure 9) (Liu C. et al., 2023) in other studies. Hence, incorporating particles provides mechanical integrity to the cell-laden construct and offers biomechanical cues and bioactivity, enhancing the BTE. More recent examples of gelatin particle systems used in bioink for BTE are listed in Table 6.

**TABLE 6:** Examples of Gelatin-particles-based bio-inks formulations for BTE.

Ink preparation	Crosslinking procedure	Structure printed	Used cells	Bioprinter	Remarks	Ref.
Bioactive glass(BaG) and Alginate, cellulose nanofibrils(CNF)	CaCl2	Square mesh	hBMSCs	3D-Bioblotter^®^	Reduced cell proliferation and viability; moreover, ALP activity improvement	Ojansivu et al. (2019)
Lanponite	UV	Mesh		BioScaffolder	Improved osteogenic differentiation, porosity, and cell viability	Cidonio et al. (2019)
CaCl_2_, SF	mushroom tyrosinase	Square mesh	hBMSCs human	3D Discovery bioprinter	Improved osteogenic potential, upregulating gene expression of BMP4, BMP2 and β-catenin. And Enhancing mineralization	Sharma et al. (2019)
Strontium	UV	Square mesh	hBMSCs	BioScaffolder	Improved cell viability and osteogenic-specific cell signal-Ling and improved printability	Alcala-Orozco et al. (2020)
Calcium silicate with strontium	UV	Square mesh	WJMSC	BioX extrusion-based 3D printer	Improved printability, tensile strength, and degradation. *In vitro* improvement of cell proliferation and differentiation as well as mineralization	Yu et al. (2020a)
Bone particles (BP)	UV	Square mesh	Primary cells of BP	3D-Bioplotter	Improved osteogenic differentiation capacity, printability, and cell viability	Ratheesh et al. (2020)
Mesoporous silica nanoparticles, along with dexamethasone and calcium phosphate	Ice cooling, UV	Disk	hBM-MSCs	3D bioprinter + Inkredible	Improved the pro-osteoconductive properties	Tavares et al. (2021)
Silanated silica	UV and cooling at 5°C–7°C	Square mesh	hBMSCs	3D bioprinter	Improved Young’s modulus and tensile strength. Also, cell proliferation and differentiation are improved	Choi et al. (2021)
HAp	UV	Cuboid crosshatch infill	MC3T3-E1	BIO-X 3D Bioprinter	Reduction of hydrogel swelling, improvement of enzymic degradation resistance, and osteogenic gene expression	Allen et al. (2022)
Nano-attapulgite	UV	Disk mesh	BMSCs and HUVECs of mouse	3D bioprinter	Improved mechanical properties and printability. Improved angiogenic activity and cell viability	Liu et al. (2023b)
α-tricalcium phosphate	UV	Helical Harversian canal and Human osseous labyrinth	ADSCs	3D Printer bio-reactor	Improvement of cell proliferation and migration, printability	Jalandhra et al. (2022)
Magnetic nanoparticles with Hap coated	UV	Disk mesh	hOBs and PDLFs of human	Pneumatic extrusion bioprinter	No toxic effect is seen, and cell migration improved	Vurat et al. (2022)
Laponite nanosilicate with alginate	CaCl_2_	Square mesh	rBMSCs of rat	Particle Cloud Bioprinter	No cytotoxicity is seen, and there is improved bone healing capability, printability, and mechanical properties	Liu et al. (2020)
Laponite, Alginate	UV, CaCl_2_	Disk mesh	BMSCs and PC12 Cells	Three-axis bioprinting system	Improvement of bone SNS activation inhibited catecholamine release from SNS and promoted bone regeneration	Li et al. (2022b)
Acetylated GM, HAp. And GM	UV	Square mesh	ASCs and HDMECs	tabletop robot TR300	Improve mechanical properties, printability, and upregulation of bone proteins FN, OPN, and Col I	Leucht et al. (2020)
Nano-HAp and alginate	CaCl_2_	Square mesh	hPDLSCs	3D Bioplotter	Improvement of the rheological properties, compressive property, and porosity and improvement of the osteogenic differentiation	Tian et al. (2021)
Alginate and nanocellulose	CaCl_2_	scaphoid	MSCs	Allevi 2 3D bioprinter	Improvement of the shape fidelity, mechanical, physicochemical properties, and osteogenic differentiation improved	Gonzalez-Fernandez et al. (2021)
Mesoporous silica nanoparticles and PEG	Temp at 11°C–22°C and UV	Square mesh	RAW264.7 and BMSCs	3D-Bioplotter	Improved printability, fidelity, and biocompatibility. Inhibition of inflammatory reactions due to BMP-4 release from M2-type macrophage	Sun et al. (2021b)
Graphene oxide and alginate	CaCl_2_	lattice-rod	hMSCs	Microextrusion bioprinter	Improved scaffold fidelity, biocompatibility, and upregulation of osteogenic-gene (PHEX, BGLAP, ALPL) expression	Zhang et al. (2021)
Hap and alginate	CaCl_2_ and UV	Square mesh	MC3T3 and HUVEC	ovoGenMMX bioprinter	Improved mechanical, printability, and cell showed enhanced osteogenic and angiogenic activities	Shahabipour et al. (2022)
Nanofibrillated cellulose, XG, β-TCP, and HAMA	UV	Square mesh	hMSCs human	BioX bioprinter	Improve viability, gel stability, mineralization capability, and upregulation of osteogenic markers OCN and RUNX2	Bedell et al. (2022)
Oxidized alginate and amine-functionalized copper (Cu)-doped mesoporous bioactive glass nanoparticles	CaCl_2_	Square mesh	HUVECs and BMSCs	3D printer	Improve printability, osteogenic differentiation cell spreading, and proliferation	Zhu et al. (2022)
Calcium silicate, along with mesoporous strontium	UV	Square mesh	WJMSC	Extrusion-based 3D printer BioX	Improvement of mechanical properties. The WJMSC osteogenic differentiation, cell differentiation, and proliferation improvement	Yu et al. (2020b)

Creating a functional and biomimetic nanocomposite bio-ink is another viable option that some researchers have taken advantage of by developing a bio-printed scaffold for orthopedic intervention. A study used (nano-silica) nSi, gelatin, and alginate to bio-print cell-laden rat bone marrow mesenchymal stem cells (rBMSCs) ECM mimicking structure. The nSi in the bio-ink improves the mechanical strength and printability of the encapsulated rBMSCs and triggers osteogenic differentiation. The *in vivo* investigation further validated the formulation’s potential for critical size defect bone repair (Liu et al., 2020). Similarly, another study created graphene oxide (GO)/alginate/gelatin hMSC-laden bio-ink to build bone-mimicking constructs via a bioprinting approach (Zhang et al., 2021).

Improving osteogenesis through neuropeptide release and neural network restoration is an appealing technique for healing large-size bone deformation. Although the defect area sympathetic nervous system (SNS) is stimulated by traumatic bone defects, causing severe catecholamine release obstructing quick bone repair. In one study (Li S. et al., 2022), nifedipine, a calcium channel blocker, was incorporated in the bio-ink to lower catecholamine concentrations in the bone defect location and promote bone regeneration. The released nifedipine restricted nerve cells’ calcium channels, preventing the activation of SNS and, eventually, reducing catecholamine production. Therefore, reducing catecholamine release facilitates an increase in the bone repair of a critical-size calvarial defect rat model by migration of BMSCs, inhibiting osteoclastogenesis *in vitro* and promoting osteogenic differentiation.

Bone tissue has a significant vascularization. The interaction of vascular and osteogenic cells is essential for developing these two very different tissue types and their physiological maintenance and repair. One study (Leucht et al., 2020) investigated an all-gelatin-based toolkit containing GM, acetylated GM (GMA), and gelatin to adjust the bio-inks characteristics toward increased printability and more significant support of vascular network creation. The co-culture of bio-printed hADSCs and human dermal microvascular endothelial cells (HDMECs) of constructs revealed tissue-specific functional cells. Interaction influenced the vascular-like architecture creation and maintenance, boosting osteogenesis. On the other hand, Shahabipour et al. (Xia et al., 2023) bio-printed an osteon-like structure by depositing osteogenic and angiogenic bio-inks from the coaxial nozzle shell and core areas. The bio-inks comprise gelatin, GM, alginate, and HAp nanoparticles with preferential HUVECs cells for the core and MC3T3 for the shell: the bio-printed coaxial structure-maintained survivability and the expression of angiogenic and osteogenic factors better than the traditional structure. Similarly, instead of HAp, amine-functionalized copper (Cu)-doped mesoporous bioactive glass nanoparticles (ACuMBGNs) were employed in another study (Zhu et al., 2022).

Because of the macrophage polarization failure and the bone defect site inflammatory milieu, large bone deformation remains a huge therapeutic problem, especially for diabetic patients. Chemicals material or chemicals with anti-inflammatory properties can get around this problem. In one of the recent studies (Sun X. et al., 2021), they combined GM, 4-arm PEG, RAW264.7 macrophages, BMSCs, and mesoporous silica nanoparticles (MSNs) loaded with BMP-4. MSNs substantially increased the mechanical strength and sustained the release of BMP-4. The released BMP-4 enhanced the polarization of RAW264.7 to M2 phase macrophages, facilitating the production of anti-inflammatory components and lower pro-inflammatory factor levels, enhancing rat model bone regeneration.

**FIGURE 10:**
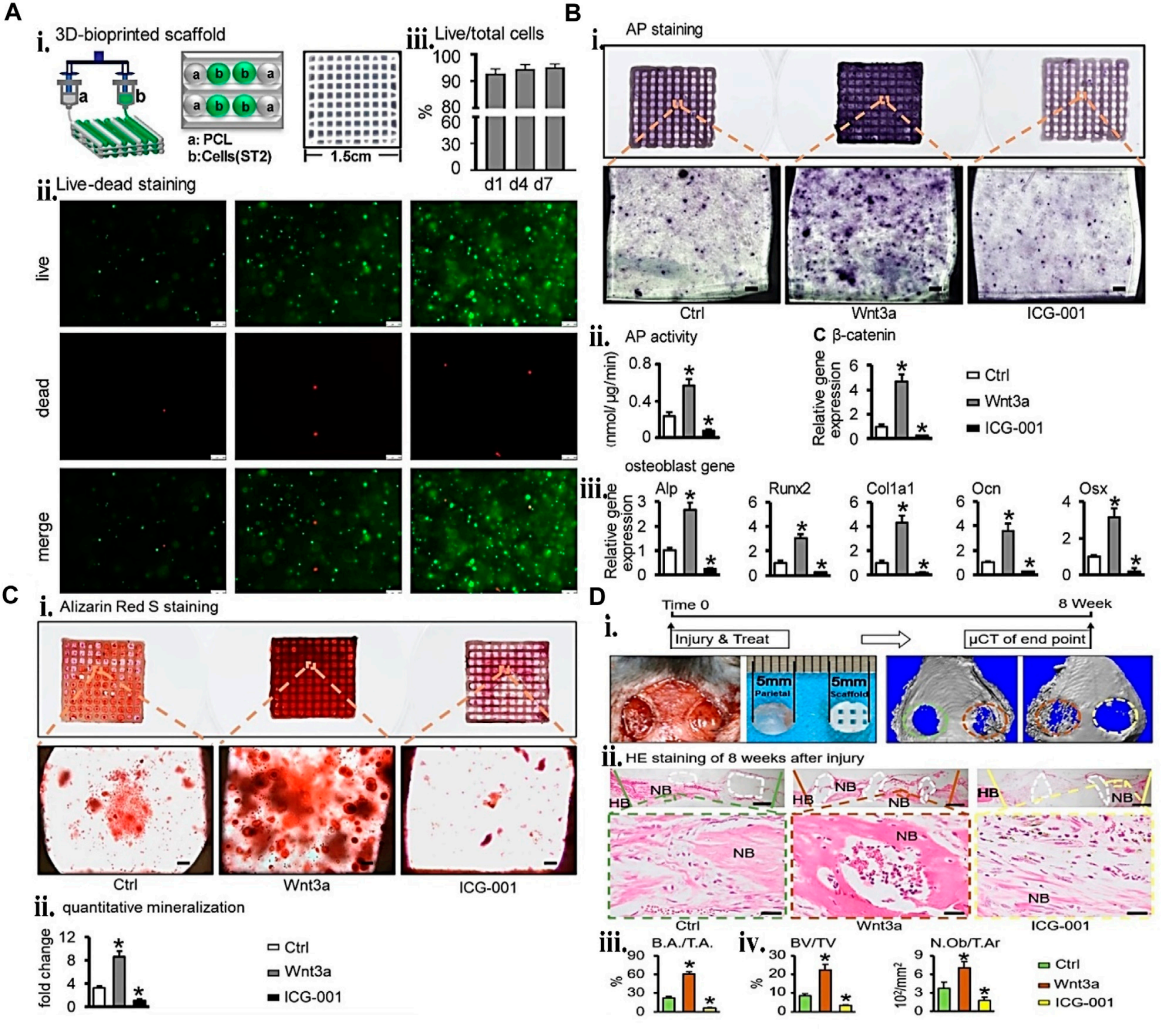
Gelatin plus polymer plastic for BTE showing **(A)** Cell viability assay and ST2/GM/PCL hybrid construct fabrication. (i) schematic representation and gross image of the 3D printed construct. (ii) live/dead staining fluorescence images. (iii) cell viability quantifications at 1,4, and 7-day points. The one-way ANOVA confirms that the cell viability was not statistically different. **(B)** Osteogenic activity of ST2/GM/PCL hybrid system. (i) ALP staining and (ii) ALP activity quantification. (iii) key factor β-catenin expression of Wnt signaling and (iv) osteogenic markers genes (Runx2, Ocn, Colla1, ALP, and Osx). **(C)** Mineralization of ST2/GM/PCL hybrid system. (i) ARS staining, (ii) mineralization quantification. **(D)** Animal study of critical-size defects in mice. (i) Micro-CT at week 8 after implantation of ST2/GM/PCL hybrid system (the control group is shown in the green circle while the brown circle depicts the Wnt3a group, and the ICG-001 group is the yellow circle). (ii) Bone defect section tissue H&E staining. The white dotted area represents PCL, and HB indicates the host bone. (iii) Bone area (B.A) per tissue area (T.A) quantitative analysis. (iv) BV/TV statistical histomorphometry analysis, Osteoblast per tissue area (N.O/T.A). Every study was compared to the control group. Reproduced with permission from Liu et al. (2022).

A hybrid system is another technique investigated for mechanically improving and creating a sturdy 3D build. This technique uses a synthetic polymer scaffold framework that meets the tissue regeneration requirements of strong mechanical characteristics, for example, orthopedic and a self-soft-regulating milieu for cells. In one study (Liu et al., 2022), GM/PCL scaffold was bio-printed and pretreated with Wnt3a loaded ST2 (bone marrow stromal cell line) for 24 h. The 24-h pretreatment increased the cell viability, proliferation, mineralization, and osteogenic differentiation of the encapsulated ST2 *in vitro* and improved osteogenesis and angiogenesis in a large-size bone defect of calvarial mice, shown in Figure 10. In a similar version, Firouzian et al. (2020) created a biomimetic rat tissue construct to simulate the heterogeneous mechanical characteristics of spinal cord tissue. The cell-laden gelatin/alginate/fibrinogen and primary rat neural cells printed in between the PLGA collagen-coated platform to mimic the soft cell tissue microenvironment. The post 14 days culture analysis of the cell viability, scaffold interface, and immunostaining indicates a homogeneous spread of stable, elongated, healthy neurites and neural cells. However, Nulty et al. (Morgan and Keaveny, 2001) formulated a fibrin-based bio-ink containing HUVEC and hBMSCs for the pre-vascularization of printed PCL scaffolds. The implanted hybrid device in rats with significant femoral bone deficiencies supports new bone formation. The *in vivo* analysis using Micro-computed tomography (CT) angiography demonstrated enhanced vascularization and large new bone formation. Hence, plastic materials do not only serve the purpose of mechanical support but also give the microenvironment of biomimetic bone tissue.

Large and open bone defects are extremely at risk of pathogens, which can result in high infection chances and delay bone healing. A scaffold with dual osteoinduction and bacterial suppression functionalities is required to promote the effective healing of infectious bone lesions. One recent study (Wang M. et al., 2021) created a hybrid system comprising modified cells using PCL/mesoporous bioactive glass/DOX and bio link. The *in vitro* and *in vivo* investigations demonstrated that the fabricated hybrid system could actively produce BMP2, which helps stimulate osteoblast development, causes ectopic bone synthesis, and has antimicrobial properties. More recent examples of gelatin hybrid systems used in bioink for CTE are listed in Table 7.

**TABLE 7:** Examples of Gelatin-hybrid-based bio-inks formulations for BTE.

Ink preparation	Crosslinking procedure	Structure printed	Used cells	Bioprinter	Remarks	Ref.
PCL, having doxycycline (DOX) and mesoporous bioactive glass (MBG) + HAMA	UV	Square network	BMSCs	3D Bioplottor	Scaffolds produced good cell viability and Antibacterial Properties	Wang et al. (2021b)
PLGA + Alginate Fibrogen	UV	Disk having a porous structure	C2C12	SLA S600	Better mechanical cell viability (14 days). Excellent immunostaining analysis data for neural cells *in vitro*	Firouzian et al. (2020)
Strontium, Magnesium, and PCL	UV	Circular and Square network	hMSCs	BioScaffolder	Better mechanical, biodegradability, bio functionality, and cell viability	Alcala-Orozco et al. (2022)
PCl, fibrin, nano-Hap (nHA), and Alginate	CaCl_2_ and UV	Cylinder having a porous structure	hBMSCs and HUVECs	Multi-head extrusion bioprinting	Improves vascularisation *in virto*	Nulty et al. (2021)
mPEG-(CL32/LA58/GA10), (PCL-ran-PLLA-ran-PGA) (PCLG) (PCLG-copolymer)	UV	Circular (Round)	MSCs	3D bioprinter	Better printability, cell viability, signal stability, and tissue formation	Kim et al. (2021)
PCL	UV	Square Network	ST2	—	Enhanced osteogenic differentiation, Proliferation, enhanced angiogenesis and osteogenesis	Liu et al. (2022)

**FIGURE 11:**
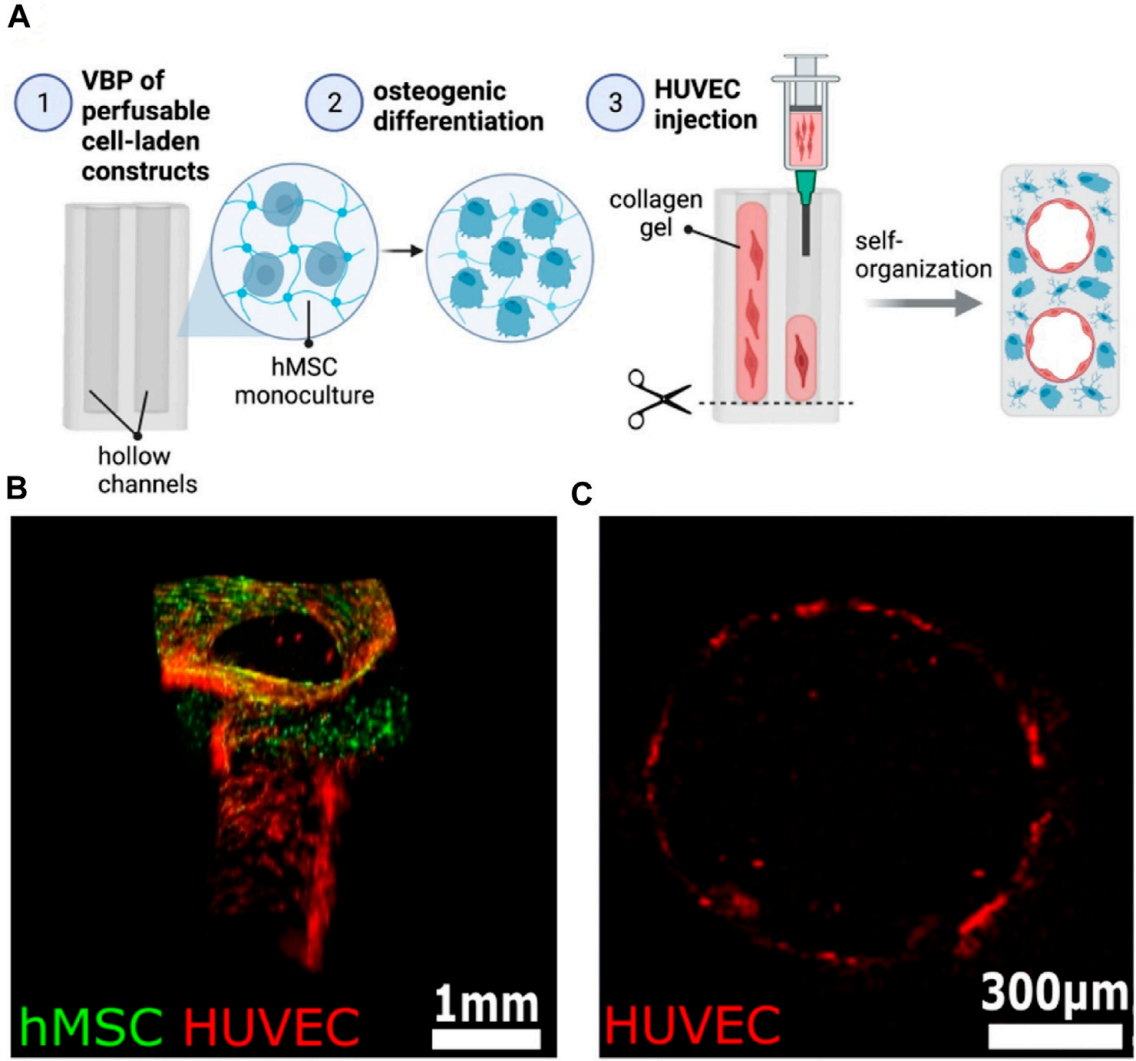
Establishment of a heterocellular perfusable pre-vascularization model. **(A)** Schematic of the experimental procedure for endothelial channel lining in 3D bioprinted constructs. **(B)** The 3D rendered confocal image of an endothelium-lined channel on day 14 demonstrates successful cell integration (hMSCs: green, HUVECs: red). **(C)** Cross-section confocal image confirms continuous endothelial lining within the channel (HUVECs: red). Scale bars: B = 1 mm, C = 300 µm. Reproduced with permission ref. Gehlen et al. (2023).

Volumetric bioprinting (VBP) has recently emerged as a revolutionary technique that utilizes light projections to fabricate centimeter-scale tissue constructs within seconds (Bernal et al., 2019; Kelly et al., 2019; Rizzo et al., 2021; Gehlen et al., 2023). This nozzle-free approach leverages existing imaging techniques like CT scans to create complex 3D structures with high resolution and exceptional cell viability. One recent study demonstrated the potential of VBP for enhanced *in vitro* bone formation using 3D endothelial co-culture (Gehlen et al., 2023). They identified a soft bioink formulation (5% GelMA, 0.05% LAP) that promotes cell-matrix interactions and communication within the 3D construct. This optimized bioink led to increased expression of bone-specific markers in co-cultured constructs compared to monocultures, suggesting accelerated osteogenic differentiation. Additionally, they successfully established a perfusable pre-vascularized bone construct (Figure 11), paving the way for future studies on bone tissue maturation and function. While promising, further research is needed to address limitations. The developed constructs exhibit limited matrix mineralization and lack mature osteocyte markers. Future studies could explore higher cell densities using optical tuning methods and incorporate additional factors like co-culture with macrophages/osteoclasts and mechanical stimulation to enhance osteogenesis (Sims and Walsh, 2012; Wittkowske et al., 2016; Bernal et al., 2022). Another work used endothelial co-culture and tomographic volumetric bioprinting (VBP) to achieve ultrafast bone tissue model bio-manufacturing. The heterocellular contacts of 3D endothelial co-cultures improve osteogenic development in printed settings. The elevated early osteocytic markers gene expression in 3D co-cultures post 3 weeks validated this osteogenic differentiation enhancement (Gehlen et al., 2022). Overall, volumetric bioprinting holds immense potential for revolutionizing BTE. Addressing the existing limitations and exploring the suggested future directions are crucial for advancing this technology toward clinical applications and creating functional bone replacements.

## 6 4D Bio-printing of gelatin-based bio-inks for orthopedic application

4D printing has evolved to counteract the shortcomings of invariability and complicated structures in tissue engineering and other bio-related disciplines, which are difficult to make via 3D printing (Li et al., 2017; Mahmood et al., 2023; Wang et al., 2023). Skylar Tibbits, an MIT professor, offered a newfangled notion at a TED (technology, entertainment, design) conference that resourcefully channeled the universe’s ingenuity in 3D toward 4D printing. As a result, an additional factor, time, must be considered in addition to the already known x, y, and z-axes geometry in 3D printing (Choi et al., 2015; Sajjad et al., 2023). The 4D printing approach allows the printed construct to vary in form (give dynamicity). Thus, it functions throughout the transformation with the help of the necessary stimuli, such as water (Sydney Gladman et al., 2016), pH (Zhang et al., 2013), thermal (Guo et al., 2018), magnetic (Kokkinis et al., 2015), and so on. Printing in 4D is rapidly expanding its bounds in almost every area, including biomimetics (Momeni and Ni, 2018), electronics (Hua et al., 2018), origami (Janbaz et al., 2016), fashion (Zarek et al., 2016), and a promising biomedical domain [devices (Zarek et al., 2017), tissue engineering (Hendrikson et al., 2017), and so on]—to investigate its dynamism.

The significant advancements in 3D and 4D printing capabilities in biomedicine have generated a subset of 3D and 4D bioprinting attributable to the actualization of physiologically suitable biopolymers involving cell incorporation. As a result, the emergence of 4D bioprinting has induced organ printing dynamicity, such as the heart and other biomedical objects, to maintain tempo with organic physiological characteristics, rendering sensitivity to the surrounding environment (Ambekar and Kandasubramanian, 2019; Rastogi and Kandasubramanian, 2019). The attribution of the 4D bio-printed construct’s responsiveness may be due to cell maturation or the biopolymer shape memory effect, which tries to instill functionalities into the bio-printed construct. Shape memory and smart materials, which have an extraordinary characteristic of storing the translation information between the parent and programmed geometry when subjected to an appropriate microenvironment (stimulus), have intrigued several scientists around the globe with therapies and medications. Nonetheless, both shape memory or smart materials and the stimulus must encourage the physiological systems functioning of the human body. Water-sourced stimulants, for instance, enrich swelling cell-laden scaffolds for varied geometries, including curving, folding, and bending, depending on the bio-printed scaffold anisotropy. Similarly, heat (close to physiological temperature) and magnetic stimuli can cause changes while sustaining cell viability. Cell maturation allows tissue creation over a long duration to mimic the natural complexities in a manufactured 3D structure for practical functioning (Korde and Kandasubramanian, 2019; Yang et al., 2019).

**FIGURE 12:**
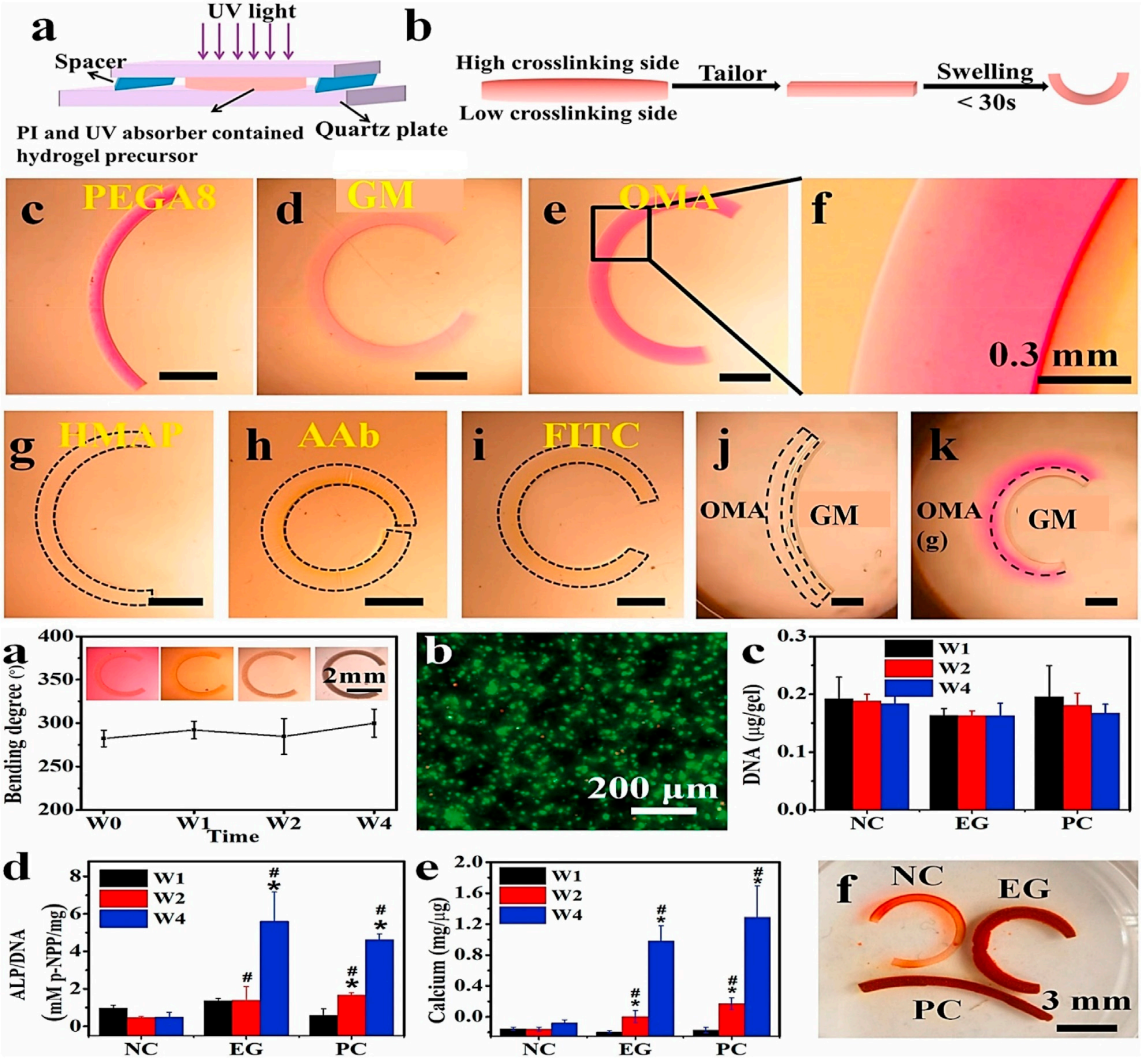
A typical gelatin-based 4D bioprinting for orthopedic application showing **(A)** hydrogel crosslinking gradient schematic, **(B)** deformation illustration of a gradient hydrogel after swelling. Curling demonstration of **(C)** PEGDA, **(D)** GM and **(E)** OMA achieved under RhB (0.03% w/v) UV absorber. **(F)** Zoom out micrograph of OMA hydrogel revealing continuous gradient; OMA curved hydrogel bar using various UV absorbers such as **(G)** FITC (0.03% w/v), **(H)** AAb (0.05% w/v), and **(I)** HMAP (0.01% w/v); bilayer hydrogel bars derived using **(J)** OMA/GM and **(K)** OMA(g)/GM illustrating the multi-material fabrication feasibility. The OMA(g) represents the OMA gradient hydrogel. Reproduced with permission from Ding et al. (2022).

According to Scopus information, despite gelatin’s value (whether in composite or pristine form), its 4D applicability in tissue regeneration and other (bio) engineering applications, such as orthopedic, remains quantitatively sparse. However, Ding et al. (2022) presented a simple method for creating a resilient and adjustable gradient via multi or single-material one-step 4D bio-fabrication (Figure 12). Various photocurable biopolymers such as GM, PEG, alginate, and their derivatives were bio-printed and examined for layer gradient degree of crosslinking with the help of a UV absorber. Furthermore, the developed simple printing strategies can be applied to other hydrogel-based applications, including ion-transfer printing, photomask-aided microfabrication, photo-patterning, and 3D bio-printing for more sophisticated construct architectures. Finally, a 4D bone-like tissue development study established proof-of-concept 4D tissue engineering.

A few years ago, the invention of 5D printing technology employed five axes to create curves, and more sophisticated things came to life. 6D printing technology now combines the ideas of 4D and 5D printing to create constructs that change geometry with time in reaction to external inputs. Future research will incorporate a mix of multi-dimensional printing technologies and intelligent materials. Multi-dimensional additive manufacturing technology will push the printing dimension to higher degrees of structural flexibility and printing efficacy, good qualities for a wide range of orthopedic applications.

## 7 Challenges in clinical translation

After scanning patients’ limbs, patient-specific 3D printing of fracture objects is becoming increasingly common. These scaffolds feature a snap closure and personalized fit, perforated and porous to increase skin visibility and reduce skin irritation to help minimize issues in the casting approach. Contrarily, overcoming the bio-printing of orthopedic tissues, many hurdles faced at the clinical stage are imperative before patient use. First and foremost, finding suitable and consistent recruitment of bio-printed cells to obtain an appropriate tissue is still challenging. In an ideal situation, the encapsulated cells must be derived or harvested from the patient to bio-print an appropriate tissue. The isolation of patient bMSCs requires the aspiration of bone marrow, followed by cell expansion in a Good Manufacturing Practices (GMP) facility and printing using a bio-ink formulation created according to GMP procedures. This technique’s intricacy is time-demanding, leading to a lag between tissue construct separation, actual implantation, and printing. As a result, those time-consuming techniques may not be therapeutically feasible when the patient requires rapid assistance.

Second, GMP facilities’ availability globally in hospitals is minimal, limiting the clinic translation to a few locations. Furthermore, the insurance company’s unwillingness to support, the added expenses, and the funding by the national health systems to make and use these products without clinical proof are a few examples of the challenges. As a result, its translatability is further limited to a few chosen medical institutes, significantly limiting the early phases of development. Finally, and probably most critically, a stumbling block exists in the research process. There are not enough surgeon scientists, tissue engineers, and scientists who can perform substantial preclinical animal testing, which serves as a prerequisite before clinical studies. In fact, several animal experiments are conducted on small animals, such as rats, thus providing minimal mechanical behavior information under physiological pressures. Increasing geometries and implant size is critical when analyzing biomaterials for human clinical translation.

Nevertheless, big-size animal models with similar features of physiological tissue stresses and implant sizes may not consistently reproduce the large joints of human biomechanics due to our upright and bipedal nature. Finally, industrial engagement is critical to translation. Due to competing interests, industrial partnerships are challenging to oversee; therefore, most academic institutions cannot handle and control the connections systematically and efficiently due to their intrinsic complexity and available resources. Even with these reservations, a significant partnership involving clinicians, fundamental research, and industry is necessary to take the developed bio-formulations from the laboratory to the bedside regularly and efficiently. As a result, in-depth animal experiments, ethical partnerships, interdisciplinary teams, and collaborations involving industries might pave the path for 3D bio-printable gelatin systems to scale through translation.

## 8 Conclusion and future prospects

Bioprinting involves the use of hydrogels and specified cells to create cell-laden constructs with the aim of engineering a particular tissue. In contrast to traditional fabrication techniques, bioprinting offers various interesting benefits, including excellent spatiotemporal resolution, desired nano/microstructures at a preferred location on the bio-printed construct, and large throughput production. The 3D bioprinting process has substantially improved printing speed, resolution, and accuracy due to rapidly emerging technologies in mechanical instruments and software. Developing a good bio-ink with appropriate biocompatibility, mechanical and rheological qualities, and printability is critical in generating a desired orthopedic cell-laden construct. Notably, gelatin is mechanically weak and cannot be stable for long; however, these two factors are important for BTE. Additionally, bioactive substances which may promote chondrogenesis and osteogenesis are also absent. As a result, gelatin and its derivative are mixed with other functional nanomaterials/polymers to develop bio-ink with adjustable characteristics.

Additionally, it is necessary to consider developing the ink to sustain various kinds of cells based on their needs. The developed bio-formulations should also support the uniform distributions of chondrocytes with the bio-printed construct and ECM repair, which is critical for CTE. Nevertheless, depending on the target site’s need, it should be able to manage the breakdown of gelatin-based scaffolds. Additionally, including various stimuli-responsive materials in bio-ink and investigating implanted cells’ response to stimuli exposure is also important.

Microfluidic networks can be printed within the scaffold to mimic blood vessels, ensuring vital nutrient and oxygen flow to implanted cells and engineered tissues. This paves the way for treating conditions like osteoporosis and fractures by printing porous scaffolds that promote bone ingrowth and faster healing. While challenges in bioink stability, long-term compatibility, and printing techniques remain, the future of 3D bioprinting with gelatin is incredibly promising. With continued research, this technology could offer personalized solutions for healing, regeneration, and, ultimately, improved quality of life for countless patients. It is worth noting that creating a commercially practical gelatin-based 3D printed product necessitates using principles from numerous fields, including software, instrumentation, biotechnology, material science, chemistry, and so on. As a result, academics worldwide with diverse skills should collaborate with the industry to create innovative bio-ink formulations on a big scale to improve patients’ quality of life.
